# Hospitalization risks associated with floods in a multi-country study

**DOI:** 10.1038/s44221-025-00425-8

**Published:** 2025-04-08

**Authors:** Zhengyu Yang, Wenzhong Huang, Joanne E. McKenzie, Rongbin Xu, Pei Yu, Yao Wu, Yanming Liu, Bo Wen, Yiwen Zhang, Wenhua Yu, Tingting Ye, Yuxi Zhang, Ke Ju, Simon Hales, Micheline de Sousa Zanotti Stagliorio Coelho, Patricia Matus, Kraichat Tantrakarnapa, Yue Leon Guo, Wissanupong Kliengchuay, Eric Lavigne, Dung Phung, Paulo Hilario Nascimento Saldiva, Yuming Guo, Shanshan Li

**Affiliations:** 1 Climate Air Quality Research Unit, School of Public Health and Preventive Medicine, Monash University, Melbourne, Victoria Australia; 2 Methods in Evidence Synthesis Unit, School of Public Health and Preventive Medicine, Monash University, Melbourne, Victoria Australia; 3 School of Life and Environmental Sciences, University of Sydney, Sydney, New South Wales Australia; 4 Department of Public Health, University of Otago, Wellington, New Zealand; 5 Department of Pathology, School of Medicine, University of São Paulo, São Paulo, Brazil; 6 School of Medicine, University of the Andes (Universidad de los Andes), Las Condes, Chile; 7 Social and Environmental Medicine, Faculty of Tropical Medicine, Mahidol University, Krung Thep Maha Nakhon, Bangkok, Thailand; 8 Environmental and Occupational Medicine, National Taiwan University College of Medicine and National Taiwan University Hospital, Taipei, Taiwan; 9 School of Epidemiology and Public Health, University of Ottawa, Ottawa, Ontario Canada; 10 Environmental Health Science and Research Bureau, Health Canada, Ottawa, Ontario Canada; 11 School of Public Health, University of Queensland, Brisbane, Queensland Australia

**Keywords:** Diseases, Health services

## Abstract

Floods of unprecedented intensity and frequency have been observed. However, evidence regarding the impacts of floods on hospitalization remains limited. Here we collected daily hospitalization counts during 2000–2019 from 747 communities in Australia, Brazil, Canada, Chile, New Zealand, Taiwan, Thailand and Vietnam. For each community, flooded days were defined as days from the start dates to the end dates of flood events. Lag–response associations between flooded day and daily hospitalization risks were estimated for each community using a quasi-Poisson regression model with a distributed lag nonlinear function. The community-specific estimates were then pooled using a random-effects meta-analysis. Based on the pooled estimates, attributable fractions of hospitalizations due to floods were calculated. We found that hospitalization risks increased and persisted for up to 210 days after flood exposure, with the overall relative risks being 1.26 (95% confidence interval 1.15–1.38) for all causes, 1.35 (1.21–1.50) for cardiovascular diseases, 1.30 (1.13–1.49) for respiratory diseases, 1.26 (1.10–1.44) for infectious diseases, 1.30 (1.17–1.45) for digestive diseases, 1.11 (0.98–1.25) for mental disorders, 1.61 (1.39–1.86) for diabetes, 1.35 (1.21–1.50) for injury, 1.34 (1.21–1.48) for cancer, 1.34 (1.20–1.50) for nervous system disorders and 1.40 (1.22–1.60) for renal diseases. The associations were modified by climate types, flood severity, age, population density and socioeconomic status. Flood exposure contributed to hospitalizations by up to 0.27% from all causes. This study revealed that flood exposure was associated with increased all-cause and ten cause-specific hospitalization risks within up to 210 days after exposure.

## Abstract (web summary)

Using 300 million hospital records from eight countries and territories, this study found that exposure to floods was associated with increased risks of hospitalizations for all causes considered: cardiovascular diseases, respiratory diseases, infectious diseases, digestive diseases, mental disorders, diabetes, injury, cancer, nervous system disorders and renal diseases.

## Main

Floods are the most frequent natural disaster, and an estimated 23% of the global population is exposed to inundation depths of >0.15 m during 1-in-100-year flood events^1^. Projections indicate an escalation in the severity, duration and frequency of floods due to the more frequent extreme precipitation events and rising sea levels due to global warming^2^. Besides the well-recognized direct health impacts caused by physical forces of floods or related accidental events (for example, drowning, electrocution and hypothermia), emerging evidence suggests that floods can indirectly have broad impacts on human health^3^. This suggests that the health impact of floods may have been underestimated and will further exacerbate as the climate changes.

Current epidemiological evidence about the health impacts of flood exposure focuses on disease incidence risks, mainly covering digestive diseases, infectious diseases and mental disorders, with some evidence for respiratory diseases, cardiovascular diseases and nervous system disorders^3–5^. However, floods can lead to risk factors that would result in health impacts beyond those covered by the current epidemiological evidence^6^. Contaminated food and water can lead to digestive diseases, while exposure to pathogens and compromised hygiene practices increases the risks of respiratory and infectious diseases. Displacement and deteriorated living environments contribute to both physical and psychological impacts. Furthermore, impaired access to healthcare and medication non-compliance (due to unavailability) can exacerbate preexisting chronic conditions. Therefore, it is plausible to hypothesize increased hospitalization risks for reasons including but not limited to cardiorespiratory diseases, mental disorders, infectious diseases and digestive diseases after flood events.

Understanding the impact of floods on hospitalization risks is crucial for healthcare providers to prepare for the increased demand of health services after flood events, for public health institutions to surveil outbreaks and for policymakers to enhance preparedness and response protocols, especially considering the anticipated surge in flood events. Despite the evident need for comprehensive knowledge, previous studies only assessed the associations of flood with a handful of cause-specific hospitalizations in a single region, including cardiovascular, respiratory and infectious diseases^7–9^. To address the knowledge gaps, we conducted a multi-country/territory study covering hospitalizations over two decades across regions with various geographical and demographic characteristics. We aimed to quantify the hospitalization risks and periods of concern of various major diseases in the aftermath of floods and to identify potential effect modifiers.

## Results

**Fig. 1:**
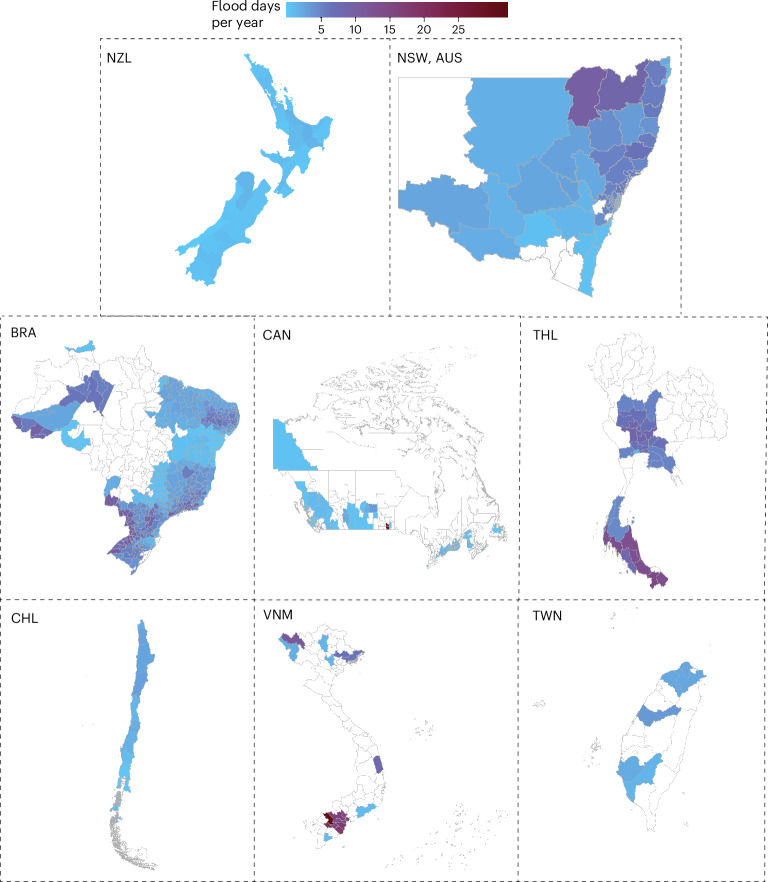
Annual flood days of the included communities during the study period. NZL, New Zealand; NSW, New South Wales; AUS, Australia; BRA, Brazil; CAN, Canada; THA, Thailand; CHL, Chile; VNM, Vietnam; TWN, Taiwan. Part of the territory may be truncated. Source data

We included 747 communities from eight countries and territories (hereafter countries/territories) in the analyses, after excluding 301 communities that were without flood events during the study period (Supplementary Table 1). Communities in the northeast region of New South Wales in Australia, along the Amazon River and the southern region of Brazil, within the Mekong Basin in Vietnam, and in the south region of Thailand experienced flood days most frequently (Fig. 1). The median length of the study period among all included communities was 13.0 (interquartile range 12.4–15.0) years (see the Supplementary Methods for details on the number of included communities, study periods and frequency of flooded days in each country or territory; Supplementary Table 1). Within 2000–2019, we included 300 million hospitalization records in the analyses (Supplementary Table 2). Supplementary Figs. 1–4 demonstrate the climate types, population density, infant mortality rate and gross domestic product (GDP) per capita of all communities.

**Fig. 2:**
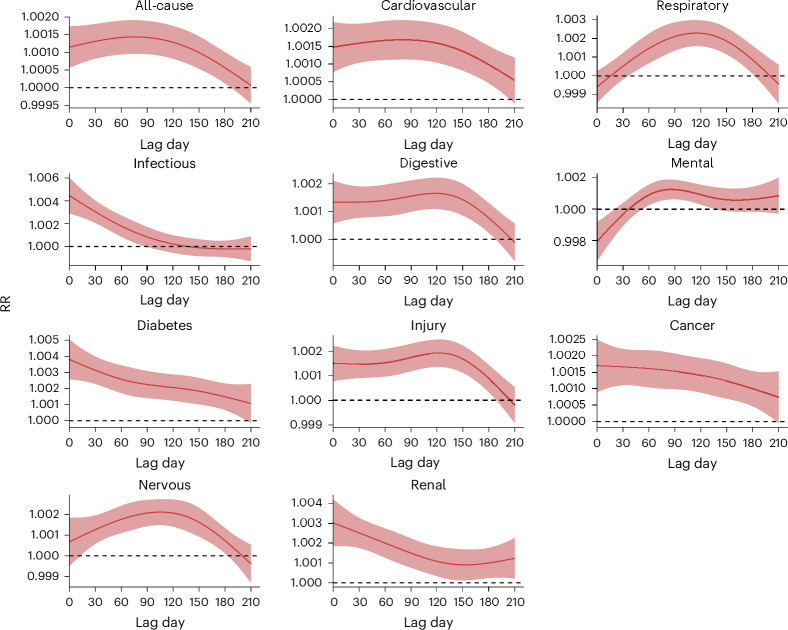
RRs of hospitalization (*n* = 300,470,192) after exposure to floods in all communities. The lines denote the estimates of RRs, and the shaded areas denote the corresponding 95% confidence intervals. Source data

**Fig. 3:**
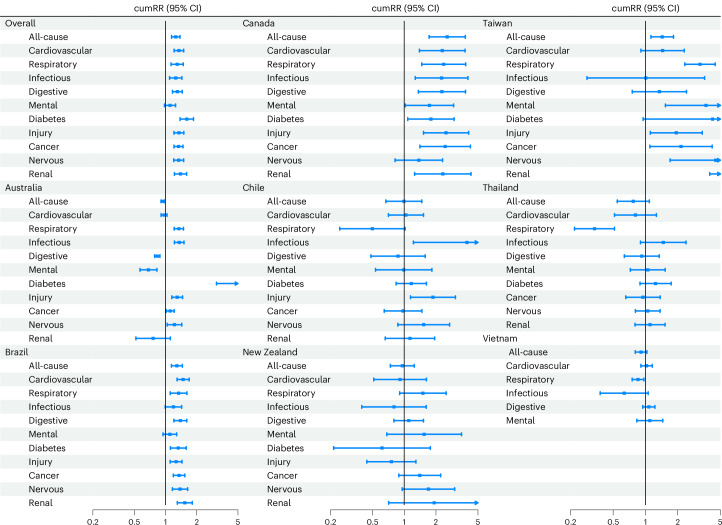
The cumRR of hospitalization (*n* = 300,470,192) in 210 days after flood exposure in all communities and by country/territory. The dots denote the estimates of RR, and the error bars represent the corresponding 95% confidence intervals (CI). Source data

Overall, we found that the risks of all-cause and cause-specific hospitalization increased after flood exposure for up to 210 days, except for hospitalizations due to infectious diseases and mental disorders, where the increases persisted for around 90 days and 150 days, respectively (Fig. 2). Most lag–response associations exhibited monotonously decreasing trends, while the associations for respiratory diseases, mental disorders and nervous system disorders displayed inverted U shapes. During 210 days after flood exposure (Fig. 3 and Supplementary Table 3), the cumulative relative risk (cumRR) of hospitalization was 1.26 (95% confidence interval 1.15–1.38) for all causes, 1.35 (1.21–1.50) for cardiovascular diseases, 1.30 (1.13–1.49) for respiratory diseases, 1.26 (1.10–1.44) for infectious diseases, 1.30 (1.17–1.45) for digestive diseases, 1.11 (0.98–1.25) for mental disorders, 1.61 (1.39–1.86) for diabetes, 1.35 (1.21–1.50) for injury, 1.34 (1.21–1.48) for cancer, 1.34 (1.20–1.50) for nervous system disorders and 1.40 (1.22–1.60) for renal diseases. The results of sensitivity analyses indicated that the estimates were robust to a range of assumptions (Supplementary Table 4).

**Extended Data Fig. 1:**
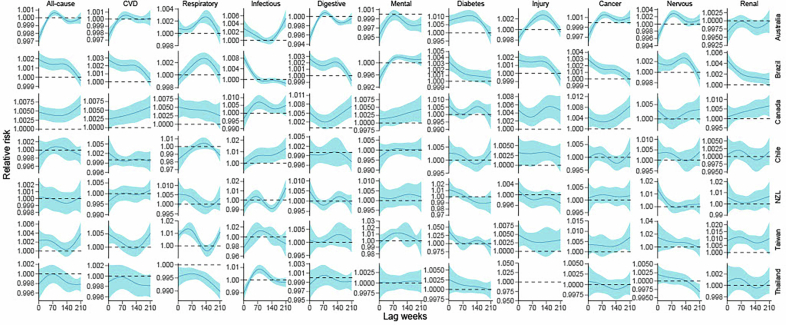
Relative risks of hospitalization (n = 300,470,192) after exposure to floods, by countries. Lines denote the estimates of relative risks, and shaded areas denote the corresponding 95% confidence intervals. Abbreviations: CVD = cardiovascular diseases; NZL = New Zealand. Source data

At the country/territory level (Fig. 3 and Supplementary Table 3), increased risks were observed for all-cause and most cause-specific hospitalizations post floods in Brazil, Canada and Taiwan. In Australia, most cause-specific hospitalization risks increased, while risks reduced for digestive diseases (cumRR 0.84, 95% confidence interval 0.79–0.89) and mental disorders (0.69, 0.57–0.83) hospitalizations, as well as for all-cause hospitalization (0.94, 0.91–0.98) after floods. The changes in hospitalization risks after floods were non-significant in New Zealand, Thailand and Vietnam, except that the hospitalization risk of respiratory diseases decreased (0.33, 0.22–0.51) after floods in Thailand. In Chile, hospitalization risks increased for infectious diseases (3.93, 1.22–12.66) and injury (1.87, 1.15–3.06). In Vietnam, the change in hospitalization risks after floods were non-significant, except for respiratory diseases (0.85, 0.75–0.97). Inconsistency of effect estimates was moderate or strong (*I*^2^ ≥ 40%) within most countries/territories (Supplementary Tables 5 and 6). Some of the country-specific lag–exposure associations (Extended Data Fig. 1) were uncertain (with wide confidence intervals), like most of the associations in Chile. Where the lag–response associations were certain, the periods of concern (relative risk (RR) >1) were mostly consistent across countries.

**Fig. 4:**
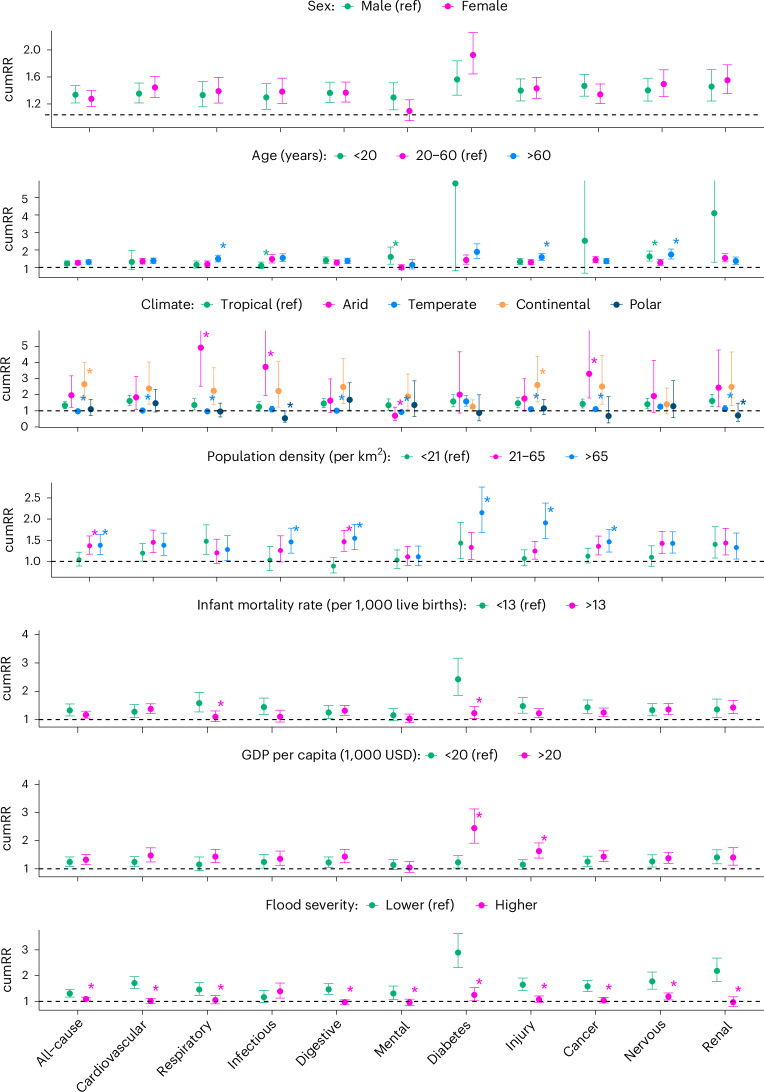
The cumRR of hospitalization (*n* = 300,470,192) in 210 days after flood exposure by potential modifiers. There was only one community (of Chile) with polar climate type. The dots denote the estimates of RR, and the error bars represent the corresponding 95% confidence intervals. *P value for difference was <0.05 for the estimate, compared with the reference group, where the comparison was performed using a meta-regression model. Detailed estimates and *P* values are presented in Supplementary Table ref, reference group; USD, constant 2011 US dollar. Source data

Results of the effect modification analyses (Fig. 4 and Supplementary Table 7) revealed that the flood–hospitalization associations varied with climate type; were weaker for more serious flood events; and were stronger among those aged <20 years or >60 years compared with the other age group, and in communities with a higher population density or a higher socioeconomic status (SES; lower infant mortality rates or higher GDP per capita). Little evidence for effect modifications by sex was found.

**Fig. 5:**
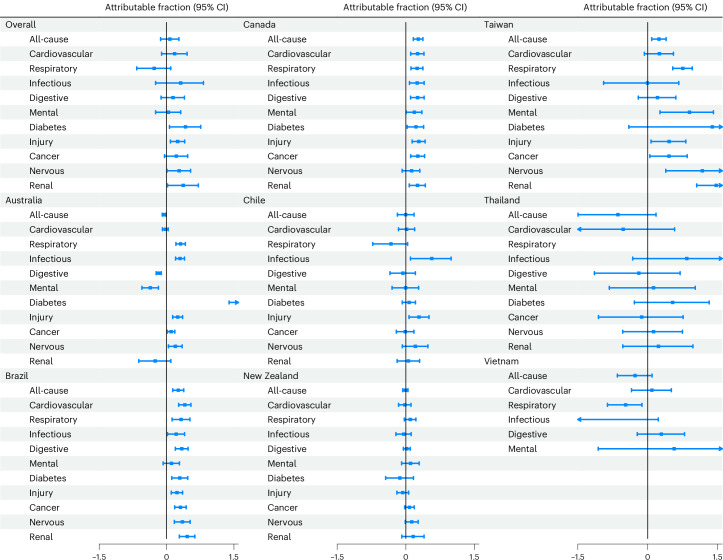
Attributable fractions (%) of hospitalizations (*n* = 300,470,192) due to flood exposure in communities impacted by floods. The dots denote the estimates of attributable fractions, and the error bars represent the corresponding 95% empirical confidence intervals. Source data

In the countries/territories included, exposure to floods contributed to hospitalizations by up to 0.27% (95% CI 0.16–0.38) from all causes, 0.41% (0.27–0.54) from cardiovascular diseases, 0.75% (0.54–0.96) from respiratory diseases, 0.57% (0.10–0.99) from infectious diseases, 0.34% (0.20–0.48) from digestive diseases, 0.90% (0.26–1.41) from mental disorders, 1.93% (1.39–2.41) from diabetes, 0.46% (0.07–0.82) from injury, 0.46% (0.05–0.85) from cancer, 1.17% (0.39–1.81) from nervous system disorders and 1.47% (1.05–1.88) from renal diseases (Fig. 5 and Supplementary Table 8). The corresponding annual numbers of hospitalization attributable to floods are presented in Supplementary Table 9.

## Discussion

Leveraging a multi-country/territory dataset spanning 2000 to 2019, our study revealed that exposure to floods was associated with increased risks of hospitalizations for all causes, cardiovascular diseases, respiratory diseases, infectious diseases, digestive diseases, mental disorders, diabetes, injury, cancer, nervous system disorders and renal diseases. Among populations exposed to floods, the risks of hospitalization increased immediately (except that respiratory diseases and mental disorders increased gradually) and returned to normal around 210 days (90 days for infectious diseases and 150 days for mental disorders) after floods. The flood–hospitalization associations varied across climate types, and they were stronger for lower-severity flood events and among those aged <20 years or >60 years compared with the other age group, and in communities with a higher population density or a higher SES. In the included countries/territories, exposure to floods contributed to hospitalizations by up to 0.27% from all causes, 0.41% from cardiovascular diseases, 0.75% from respiratory diseases, 0.57% from infectious diseases, 0.34% from digestive diseases, 0.90% from mental disorders, 1.93% from diabetes, 0.46% from injury, 0.46% from cancer, 1.17% from nervous disorders and 1.47% from renal diseases.

### Associations between floods and hospitalization

We noted that some studies had quantified the association between flood and hospitalization risk. One study observed an increased risk of hospitalization due to schizophrenia during 5–15 days after floods in a provincial capital city of mainland China in 2005–2014 (ref. ^9^). In addition, the results indicated that the association was stronger among those aged <40 years. Another study in seven provinces in the Vietnam Mekong River Delta found that the risks of hospitalizations for non-external causes (RR 1.07, 95% confidence interval 1.03–1.11), infectious diseases (1.16, 1.04–1.30) and respiratory diseases (1.26, 1.16–1.36) increased during and after flood seasons in 2011–2014 (ref. ^8^). The increase in hospitalization risk lasted for around 4 weeks for non-external causes and infectious diseases, and more than 4 weeks for respiratory diseases. The other study observed that flood exposure was associated with increased hospitalization risks of cardiovascular diseases (1.04, 1.02–1.06), chronic respiratory diseases (1.07, 1.02–1.11), respiratory infections (1.11, 1.05–1.17) and food- or water-borne diseases (1.17, 1.06–1.30) in New York State during 2002–2018 (ref. ^7^). The findings of these studies generally align with our findings, but differences exist in association strengths and maximum lag periods. The differences may stem from variations in spatial coverage and exposure assessment methods. Our study notably extended the spatial coverage to eight countries/territories, while previous studies were limited to a city or a small region; the study in mainland China defined flood events with cumulative rainfall over consecutive days, which may result in exposure misclassification because rainfall is only one of the factors leading to flood occurrences.

Our study identified increases in hospitalization risks of all-cause and cause-specific diseases in 210 days after flood exposure. There are several potential pathways, which may explain the adverse and lasting health effects. One way is through the contamination of water supply system^10^, which can elevate the risk of digestive diseases and aid the spread of infectious diseases^11^. In addition, floods can create environments that are conducive to the growth of fungi, bacteria, viruses and vectors such as mice, cockroaches and mosquitoes^12^. These environments can trigger outbreaks of respiratory, digestive and infectious diseases^12^. Floods may also force massive evacuations, causing displacement. Even when temporary shelters are provided, insufficient sanitation facilities often result in hygiene issues, increasing the likelihood of respiratory, digestive and infectious diseases^6^. Furthermore, access and capacity healthcare services may be impaired after floods, leading to delays in regular medical interventions, which include dialysis for renal diseases, chemotherapy and radiotherapy for cancer, and medication regimens for cardiovascular diseases, respiratory diseases, infectious diseases, digestive diseases, mental disorders, diabetes, nervous system disorders and renal diseases. Exposure to environmental hazards, such as extreme temperatures, high humidity and indoor air pollution, could contribute to a broad range of diseases^13–16^. Finally, long-term psychological stress (for example, from property damage and financial losses) can worsen or induce adverse health outcomes by compromising the immune system, disrupting sleep, leading to substance abuse and diminishing self-care^17,18^.

We found that the RR of hospitalization for mental disorders was lower than for the other diseases. This could be due to several reasons. First, the onsets of mental disorders were less immediately compared with the other diseases^4^. This is supported by the observed lag–response associations. Second, some individuals might be reluctant to seek help for mental health concerns because of stigma^19^. Third, the other diseases (for example, injury and infectious diseases) might have been prioritized, so the hospitalization demands of mental disorders were less fulfilled in the 210 days after floods.

Our results also revealed counterintuitive findings, with a reduction or no difference in the risk of all-cause hospitalization after floods in certain countries/territories (for example, respiratory diseases in Thailand). These findings might be attributed to impaired admission capacity of and access (that is, traffic system) to local hospitals, implementation of protective public health interventions, or raised awareness of health after floods. In Australia, the hospitalization risks of some cause-specific diseases decreased after floods, while the hospitalization risks of other cause-specific diseases increased after floods. A reason for this might be that the hospitalizations of some diseases were prioritized after floods, so the hospitalizations of other diseases were delayed and limited in number. Further research is warranted to reveal the underlying mechanisms.

### Effect modification

Our findings align with the findings reported by the US Global Change Research Program, indicating that children or adolescents and older adults are particularly vulnerable to various health impacts of natural disasters^20^. Children or adolescents are more vulnerable because some of their organs are still developing and they take in more food, water and air per unit of body weight compared with adults, which may expose them to more hazards during and after floods^21,22^. A cross-national analysis of population surveys from 29 countries and a large-scale meta-analysis of 192 epidemiological studies both indicated that the peak age of the first onset of mental disorder is around 14 years (refs. ^23,24^). Older adults are generally more frail, with existing illness, or dependent on nursing or assisted living facilities, and their compromised mobility exposes them to more environmental hazards during and after floods^25^. Our findings suggest that local climate type modifies the association between flood and hospitalization risks, which may be explained by several potential reasons. First, the infrastructure (for example, traffic and sewage) and health system resilience and preparedness (for example, food and water) to floods might vary with climate type. Second, the population vulnerability and prevalence of diseases might be different among communities with different climate types^26^. Communities with high population density exhibited stronger associations, possibly due to the longer time of exposure to hazardous environments caused by congestion during evacuations^27^; abundant medical sources that were able to admit more patients; and a higher likelihood of infectious disease outbreaks^6^.

Counterintuitively, the flood–hospitalization associations showed less harm in communities with a lower SES (compared with a higher SES), where the residents were often thought to be more vulnerable to health hazards. The reason for this might be that a lower SES generally indicated less medical resources and resilience to natural disasters, resulting in hospitalization requests not being fulfilled owing to hospitals reaching capacity sooner after floods. In addition, people in this community might die before arriving at hospitals or might be unable to travel to hospital owing to traffic infrastructure interruption during and after floods, compared with people in communities where the traffic infrastructures were resilient to flooding disasters. Consequently, communities with a lower SES presented a smaller increase in hospitalization risk compared with communities with a higher SES.

Surprisingly, the effects of more serious floods on hospitalization risks were less harmful. This is an unusual finding because more serious floods generally have a greater impact on the environment and infrastructure. One possible reason is that more serious floods had a greater impact on the capacities of healthcare systems and the traffic systems, leading to more patients not being admitted to hospital or dying before admission. Another possible reason is that serious floods forced large-scale evacuations, and individuals changed their residential community, so the hospitalizations among these individuals were excluded from the hospitalization of the original community.

### Clinical and policy implications

Our findings have important implications for clinical and policy decisions. Health service providers should anticipate increased health risks during and after floods and prepare for the heightened service demands, possibly through strengthening capacities in medical supplies, human resource management and triage strategy. Public health institutions should closely monitor reasons for hospitalization after floods as a method for disease control and efficient resource allocation in the aftermath of floods. Policymakers should prioritize enhancing health system resilience to natural disasters, recognizing that overwhelmed health systems after floods can lead to severe disease burden and even avoidable deaths. Moreover, policymakers should also improve disaster preparedness, early warning and detection systems, and efficient disaster response protocols to reduce the hazardous exposure during and after floods.

Our findings highlight the importance of improving the resilience to climate change. Healthcare systems and traffic infrastructure that are resilient to flooding disasters can ensure that hospitalization needs are fulfilled during and after the disasters, reducing avoidable deaths and lessening the health burden. Additional attention should be given to communities with a low SES status, especially to those in low-income countries, given that the residents in these countries are exposed to more floods and have weaker abilities to migrate out of these flooding areas^28^.

### Strengths and limitations

We included communities with diverse characteristics (for example, climate, geographic, demographic and socioeconomic) and all flood events in these communities. In this way, we limited major factors that may compromise the generalizability of our findings^29^. By estimating the impacts on all-cause and ten cause-specific hospitalizations, this study provides comprehensive insight into the health impacts of exposure to floods.

This study has some limitations. The Dartmouth Flood Observatory (DFO) dataset primarily covers flood events mentioned in the news and may underrepresent floods, especially in South America^2^. This might have resulted in exposure misclassification, where flooded days were regarded as non-flooded days. However, this would only bias the associations towards the null, and the extent of the underrepresentation of floods was small^2^. Besides, the exposure assessment was at the community level rather than at the individual level because exact residential addresses were confidential. Some people might reside in non-flooded areas in a community that was deemed as flooded. However, these people would still be impacted by floods through interruptions to infrastructure and health services, impairment of food and water supply systems, evacuations and psychological stresses, and the exposure misclassification would probably bias our estimates towards the null. Furthermore, it is important to note that the observed increases in hospitalizations do not equate to the increases in hospitalization demands because some cause-specific hospitalizations may be low priority, the admission capacity of hospitalizations can be impaired, and the access to healthcare can be interrupted. However, this would lead to underestimation of the hospitalization risks.

## Conclusions

This study fills a gap in the knowledge about the associations between floods and hospitalization risks, based on a multi-country/territory dataset and standard time-series statistical methods. We found that flood exposure was associated with increased hospitalizations of various causes within up to 210 days after exposure; the associations were modified by climate type and flood severity, and were stronger among those aged <20 years or >60 years compared with the other age group, and in communities with a higher population density or a higher SES. Policymakers and health professionals should raise awareness of the increased hospitalization demands from a broad range of diseases after floods to improve disaster response strategies and health system resilience to optimize the prognosis of the incidence or onset of diseases during and after floods to cope with the health challenges brought by climate change.

## Methods

### Hospitalization data

Hospitalization data were obtained from the authorities of each country/territory. These data well represented all hospitalizations across New South Wales (containing 32% of the population in Australia), Brazil, Canada, Chile, New Zealand, Taiwan, Thailand and Vietnam of differing periods between 2000 and 2019. Details of the data sources are provided in the Supplementary Methods. Daily numbers were calculated for hospitalizations from all causes, cancer (International Classification of Diseases code C00-97), cardiovascular diseases (I00-99 and G45-46), diabetes (E10-14), digestive diseases (K00-93), infectious diseases (A00-99 and B00-99), injury (S00-99 and T00-98), mental disorders (F00-99 and R40-46), nervous system disorders (G00-99), renal diseases (N00-19) and respiratory diseases (J00-99). For each hospitalization record, we obtained sex, age group (<20, 20–60, >60 years; not available for records in Vietnam), admission date and community of residence of the patient (see Supplementary Table 1 for official names of the communities in each country/territory). The records of diabetes, injury, cancer, nervous system disorders and renal diseases were not available for Vietnam, and the records of injury were not available for Thailand.

### Flood exposure

We retrieved flood events data during 2000–2019 from the DFO^30^. This is a global flood catalogue that well represents global flood events over time and has been validated and used for flood exposure assessment^2,29^. For each flood event, point and areal locations representing flooded surfaces were accumulated through news, governments and instruments. The flooded area for each event was defined as the polygon that bounded all of these points and areas. The polygons were validated by satellite observations using the MODIS (Moderate Resolution Imaging Spectroradiometer) sensors, whose spatial resolution was 250 m (ref. ^31^). Using the spatial estimates of flooded areas in the DFO, we determined that a community was flooded if its centroid was within the flooded areas. In the DFO, start dates (that is, when floods were officially recognized), end dates (that is, when floods were officially announced receding) and severities (lower: large or very large (return period <100 years); higher: extreme (return period ≥100 years)) of flood events were consistently defined across countries^32^. For each flooded community, we defined the exposure, flooded days, as days from the start dates to the end dates of flood events. In occasions that a community was impacted by two floods, we categorized the community as being impacted by the flood with a higher severity.

### Meteorological data

Population-weighted daily mean temperatures of each community were calculated on the basis of population data sourced from Landscan (https://landscan.ornl.gov/) and temperature and precipitation data collected from the ERA5-Land dataset^33^, which provided hourly observations of global air temperature at 2 m above ground and precipitation at a spatial resolution of 0.25° (28 km).

### Statistical analysis

#### Two-stage analytical approach

To estimate the associations between flood exposure and hospitalization, we used a two-stage analytical approach, which was outlined in previous studies^29,34,35^. In the first stage, a quasi-Poisson regression model in combination with a distributed lag nonlinear function was used to estimate the lag–response association between flood exposure and hospitalization risk over 0–210 lag days (that is, 0–210 days after exposure) for each community. The maximum lag period of 210 days was selected because a previous study and our preliminary results suggested that the impact of flood exposure on hospitalization risk lasted for around 7 months (ref. ^36^). To model the lag–response association, a cross-basis function was used. Each cross-basis function consisted of two dimensions that defined the exposure–response and the lag–response associations, respectively. The lag–response association describes the temporal change in risk over the maximum lag period after exposure^37^. Specifically, the exposure–response association was modelled with a strata function (strata: not exposed versus exposed), and the lag–response association was modelled with a natural cubic spline with four degrees of freedom. To control confounding effects within the model, we included a cubic B-spline with three degrees of freedom for date to model long-term trends, a cyclic cubic B-spline with three equally spaced knots for day of the year to model seasonal trends, and an indicator for day of the week^38^. In addition, we respectively controlled the potential confounding effects introduced by temperature and precipitation through a cross-basis function of daily mean temperature with a maximum lag period of 21 days and a cross-basis function of daily precipitation with a maximum lag period of 14 days, as suggested by previous studies^16,39^. Detailed information about the community-specific model is provided in the Supplementary Methods. The community-specific associations between flood and hospitalization risk were estimated in the general population, as well as in different sexes (female and male) and age groups (<20, 20–60 and >60 years old). Communities not experiencing any flood events during the study period were excluded.

In the second stage, overall lag–response associations between floods and hospitalization risks were estimated by pooling the coefficients and covariance matrixes of the cross-basis functions that quantified the community-specific associations, using random-effects multivariate meta-analyses with restricted maximum likelihood estimation. The overall lag–response associations were estimated for all communities and for each country/territory, climate type (Köppen climate classification), level of population density (cut-off points: tertiles), infant mortality rate (lower versus higher than median) and gross domestic product (GDP) per capita (lower versus higher than median)^35^. Details of the data sources and assessments of population density, infant mortality rate and GDP per capita for each community are available in the Supplementary Methods. For each meta-analysis, we quantified inconsistency with the *I*^2^ statistic and tested for heterogeneity using Cochran’s *Q* test. With each lag–response association, a cumRR was calculated by cumulating the RRs of all lag days over the maximum lag period^40^. Sensitivity analyses were performed to examine the robustness of the results (Supplementary Methods).

#### Effect modifications

To identify potential effect modifiers for the flood–hospitalization associations, the cumRRs across different strata of potential modifiers (sex and age groups, climate types, population density and SES variables (infant mortality rate and GDP per capita)) were compared using random-effects meta-regressions (because these estimates were based on different populations)^41^. To assess the effect modification by flood severity, we first estimated the effects of different flood severities by modifying the exposure strata function from two strata to three strata (that is, not exposed, exposed to lower-severity floods, and exposed to higher-severity floods) in the first stage. After calculating the cumRRs of different flood severities, fixed-effect meta-regressions were used to test whether the effect estimates were modified by flood severity (because these estimates were based on the same or overlapping populations)^41^.

#### Attributable fraction

We calculated the attributable fraction of hospitalizations due to floods for each country/territory on the basis of the country-/territory-level associations estimated at the second stage^13^. The specific method and formulas used are presented in the Supplementary Methods.

For all data analyses, we used R software (version 4.1.1). To perform the two-stage analytical approach, we used the packages dlnm and mixmeta^42^.

### Ethics declarations

This study was approved by the Monash University Human Research Ethics Committee (ID 24439). The authors confirm that the original data collection was compliant with ethics and legal frameworks (local and international), including regarding consent and privacy.

## Supplementary information

Supplementary Information Supplementary methods, Tables 1–9 and Figs. 1–4.

## Source data

Source Data Fig. 1 Unprocessed western blots and/or gels.

Source Data Fig. 2 Unprocessed western blots and/or gels.

Source Data Fig. 3 Statistical source data.

Source Data Fig. 4 Statistical source data.

Source Data Fig. 5 Statistical source data.

Source Data Extended Data Fig. 1 Unprocessed western blots and/or gels.

## Data Availability

According to the agreement with the data custodians, the hospitalization data cannot be shared. However, the data are available upon sending application to the data custodians specified in the Supplementary Methods. The Dartmouth Flood Observatory is publicly available at https://floodobservatory.colorado.edu/. Population data were sourced from Landscan at https://landscan.ornl.gov/. Data of weather predictors are open access and are available at https://cds.climate.copernicus.eu/datasets/reanalysis-era5-land?tab=overview. Infant mortality rate data are available at https://sedac.ciesin.columbia.edu/data/set/povmap-grdi-v1. Gross domestic product data were collected from https://datadryad.org/stash/dataset/doi:10.5061/dryad.dk1j0.

## Code availability

The codes that support the findings of this study are openly available via GitHub at https://github.com/ZhengyuEddy/FloodHospitalization.

## References

[1] Rentschler, J., Salhab, M. & Jafino, B. A. Flood exposure and poverty in 188 countries. *Nat. Commun.* **13**, 3527 (2022). 35764617 10.1038/s41467-022-30727-4 PMC9240081

[2] Tellman, B. et al. Satellite imaging reveals increased proportion of population exposed to floods. *Nature* **596**, 80–86 (2021). 34349288 10.1038/s41586-021-03695-w

[3] Yang, Z. et al. Mortality and morbidity risks associated with floods: a systematic review and meta-analysis. *Environ. Res.*10.1016/j.envres.2024.120263 (2024). 10.1016/j.envres.2024.120263 39481788

[4] Zhong, S. et al. The long-term physical and psychological health impacts of flooding: a systematic mapping. *Sci. Total Environ.* **626**, 165–194 (2018). 29339262 10.1016/j.scitotenv.2018.01.041

[5] Flores, A. B., Sullivan, J. A., Yu, Y. & Friedrich, H. K. Health disparities in the aftermath of flood events: a review of physical and mental health outcomes with methodological considerations in the USA. *Curr. Environ. Health Rep.* **11**, 238–254 (2024). 38605256 10.1007/s40572-024-00446-7

[6] Paterson, D. L., Wright, H. & Harris, P. N. A. Health risks of flood disasters. *Clin. Infect. Dis.* **67**, 1450–1454 (2018). 30986298 10.1093/cid/ciy227

[7] Deng, X. et al. The independent and synergistic impacts of power outages and floods on hospital admissions for multiple diseases. *Sci. Total Environ.* **828**, 154305 (2022). 35257771 10.1016/j.scitotenv.2022.154305 PMC12184828

[8] Phung, D. T., Warren, J. L., Chu, C. M.-Y. & Dubrow, R. Relationship between flood severity and risk of hospitalisation in the Mekong River Delta of Vietnam. *Occup. Environ. Med.* **78**, 676–678 (2021). 34282039 10.1136/oemed-2021-107768

[9] Wei, Q. et al. Association between floods and hospital admissions for schizophrenia in Hefei, China: the lag effects of degrees of floods and time variation. *Sci. Total Environ.* **698**, 134179 (2020). 31514040 10.1016/j.scitotenv.2019.134179

[10] Barbetta, S. et al. Assessment of flooding impact on water supply systems: a comprehensive approach based on DSS. *Water Resour. Manage.* **36**, 5443–5459 (2022).

[11] Lee, J., Perera, D., Glickman, T. & Taing, L. Water-related disasters and their health impacts: a global review. *Prog. Disaster Sci.* **8**, 100123 (2020).

[12] Suhr, F. & Steinert, J. I. Epidemiology of floods in sub-Saharan Africa: a systematic review of health outcomes. *BMC Public Health* **22**, 268 (2022). 35144560 10.1186/s12889-022-12584-4 PMC8830087

[13] Chen, G. et al. Mortality risk attributable to wildfire-related PM2·5 pollution: a global time series study in 749 locations. *Lancet Planet. Health* **5**, e579–e587 (2021). 34508679 10.1016/S2542-5196(21)00200-X

[14] Zhao, Q. et al. Global, regional, and national burden of mortality associated with non-optimal ambient temperatures from 2000 to 2019: a three-stage modelling study. *Lancet Planet. Health* **5**, e415–e425 (2021). 34245712 10.1016/S2542-5196(21)00081-4

[15] Wu, Y. et al. Global, regional, and national burden of mortality associated with short-term temperature variability from 2000–19: a three-stage modelling study. *Lancet Planet. Health* **6**, e410–e421 (2022). 35550080 10.1016/S2542-5196(22)00073-0 PMC9177161

[16] He, C. et al. Rainfall events and daily mortality across 645 global locations: two stage time series analysis. *Br. Med. J.* **387**, e080944 (2024). 39384295 10.1136/bmj-2024-080944 PMC12036573

[17] Trief, P. M., Ouimette, P., Wade, M., Shanahan, P. & Weinstock, R. S. Post-traumatic stress disorder and diabetes: co-morbidity and outcomes in a male veterans sample. *J. Behav. Med.* **29**, 411–418 (2006). 16865552 10.1007/s10865-006-9067-2

[18] Sharpe, I. & Davison, C. M. Climate change, climate-related disasters and mental disorder in low- and middle-income countries: a scoping review. *BMJ Open* **11**, e051908 (2021). 34649848 10.1136/bmjopen-2021-051908 PMC8522671

[19] Volkow, N. D., Gordon, J. A. & Koob, G. F. Choosing appropriate language to reduce the stigma around mental illness and substance use disorders. *Neuropsychopharmacology* **46**, 2230–2232 (2021). 34276051 10.1038/s41386-021-01069-4 PMC8580983

[20] Gamble, J. L. et al. in *The Impacts of Climate Change on Human Health in the United States: A Scientific Assessment* Ch. 9, 247–286 (US Global Change Research Program, 2016); 10.7930/J0Q81B0T

[21] Zheng, L. Y., Sanders, A. P., Saland, J. M., Wright, R. O. & Arora, M. Environmental exposures and pediatric kidney function and disease: a systematic review. *Environ. Res.* **158**, 625–648 (2017). 28727988 10.1016/j.envres.2017.06.029 PMC5821495

[22] Rauh, V. A. & Margolis, A. E. Research review: environmental exposures, neurodevelopment, and child mental health—new paradigms for the study of brain and behavioral effects. *Child Psychol. Psychiatry* **57**, 775–793 (2016). 10.1111/jcpp.12537 PMC4914412 26987761

[23] Patten, R. V. et al. Cognitive performance in functional seizures compared with epilepsy and healthy controls: a systematic review and meta analysis. *Lancet Psychiatry* **11**, 516–525 (2024). 38879275 10.1016/S2215-0366(24)00132-9 PMC13742074

[24] Solmi, M. et al. Age at onset of mental disorders worldwide: large-scale meta-analysis of 192 epidemiological studies. *Mol. Psychiatry* **27**, 281–295 (2022). 34079068 10.1038/s41380-021-01161-7 PMC8960395

[25] Bell, J. E. et al. in *The Impacts of Climate Change on Human Health in the United States: A Scientific Assessment* Ch. 4, 99–128 (US Global Change Research Program, 2016); 10.7930/J0BZ63ZV

[26] Mora, C. et al. Over half of known human pathogenic diseases can be aggravated by climate change. *Nat. Clim. Chang.* **12**, 869–875 (2022). 35968032 10.1038/s41558-022-01426-1 PMC9362357

[27] Wang, Z., Huang, J., Wang, H., Kang, J. & Cao, W. Analysis of flood evacuation process in vulnerable community with mutual aid mechanism: an agent-based simulation framework. *IJERPH* **17**, 560 (2020). 31952331 10.3390/ijerph17020560 PMC7013711

[28] McDermott, T. K. J. Global exposure to flood risk and poverty. *Nat. Commun.* **13**, 3529 (2022). 35764615 10.1038/s41467-022-30725-6 PMC9239995

[29] Yang, Z. et al. Mortality risks associated with floods in 761 communities worldwide: time series study. *BMJ*10.1136/bmj-2023-075081 (2023). 10.1136/bmj-2023-075081 PMC10548259 37793693

[30] Brakenridge, G. R. Global active archive of large flood events. *Dartmouth Flood Observatory* http://floodobservatory.colorado.edu/ (accessed 31 May 2023).

[31] Carozza, D. A. & Boudreault, M. A global flood risk modeling framework built with climate models and machine learning. *J. Adv. Model. Earth Syst.* **13**, e2020MS002221 (2021).

[32] Kundzewicz, Z. W., Pińskwar, I. & Brakenridge, G. R. Large floods in Europe, 1985–2009. *Hydrol. Sci. J.* **58**, 1–7 (2013).

[33] Muñoz-Sabater, J. et al. ERA5-Land: a state-of-the-art global reanalysis dataset for land applications. *Earth Syst. Sci. Data* **13**, 4349–4383 (2021).

[34] Bhaskaran, K., Gasparrini, A., Hajat, S., Smeeth, L. & Armstrong, B. Time series regression studies in environmental epidemiology. *Int. J. Epidemiol.* **42**, 1187–1195 (2013). 23760528 10.1093/ije/dyt092 PMC3780998

[35] Sera, F. & Gasparrini, A. Extended two-stage designs for environmental research. *Environ. Health Glob.* **21**, 41 (2022). 10.1186/s12940-022-00853-z PMC9017054 35436963

[36] Miyamori, D. et al. How the 2018 Japan floods impacted nursing home admissions for older persons: a longitudinal study using the long-term care insurance comprehensive database. *J. Am. Med. Dir. Assoc.* **24**, 368–375 (2023). 36587929 10.1016/j.jamda.2022.11.021

[37] Gasparrini, A. Modeling exposure–lag–response associations with distributed lag non-linear models. *Stat. Med.* **33**, 881–899 (2014). 24027094 10.1002/sim.5963 PMC4098103

[38] Scortichini, M. et al. Excess mortality during the COVID-19 outbreak in Italy: a two-stage interrupted time-series analysis. *Int. J. Epidemiol.* **49**, 1909–1917 (2020). 10.1093/ije/dyaa169 PMC7665549 33053172

[39] Gasparrini, A. et al. Mortality risk attributable to high and low ambient temperature: a multicountry observational study. *Lancet* **386**, 369–375 (2015). 26003380 10.1016/S0140-6736(14)62114-0 PMC4521077

[40] Gasparrini, A. & Leone, M. Attributable risk from distributed lag models. *BMC Med. Res. Methodol.* **14**, 55 (2014). 24758509 10.1186/1471-2288-14-55 PMC4021419

[41] Borenstein, M., Hedges, L. V., Higgins, J. P. T. & Rothstein, H. R. A basic introduction to fixed-effect and random-effects models for meta-analysis. *Res. Synth. Methods* **1**, 97–111 (2010). 26061376 10.1002/jrsm.12

[42] Sera, F., Armstrong, B., Blangiardo, M. & Gasparrini, A. An extended mixed‐effects framework for meta‐analysis. *Stat. Med.* **38**, 5429–5444 (2019). 31647135 10.1002/sim.8362

