# Supplementary material for: Hospitalization risks associated with floods in a multi-country study
Source: Nat Water. 2025 Apr 8;3(5):561–70. doi: 10.1038/s44221-025-00425-8 (PMC12098117; doi:10.1038/s44221-025-00425-8)
Supplement: Supplementary file 1 — Supplementary methods, Tables 1–9 and Figs. 1–4. [file 44221_2025_425_MOESM1_ESM.pdf]

nature water

Supplementary information https://doi.org/10.1038/s44221-025-00425-8

Hospitalization risks associated with floods in a multi-country study

In the format provided by the authors and unedited

Supplementary Appendix

Table of Contents

1. Methods 2

1.1 Hospitalization data 2

1.2 Demographic and socioeconomic status variables 2

1.3 Community-specific model 3

1.4 Sensitivity analyses 3

1.5 Calculation of attributable fraction 4

2. Results 5

Table S1. Characteristics of the communities in each country/territory 5

Table S2. Number of hospitalizations in all communities during the study period and by country/territory. 6

Table S3. Cumulative relative risks with 95% confidence interval of hospitalization in 210 days post flood exposure in all communities and by country/territory. 7

Table S4. Results of sensitivity analyses changing maximum lag period and degrees of freedom of lag-response association. 8

Table S5. I^2 statistics of pooled effect estimates among communities. 9

Table S6. Heterogeneities of effect estimates among all communities and communities in each country/territory and by potential modifiers. 11

Table S7. Cumulative relative risks of hospitalization in 210 days post flood exposure by potential modifiers. 13

Table S8. Attributable fractions (%) of hospitalizations due to flood exposure in communities impacted by floods. 15

Table S9. Annual flood-attributable hospitalizations in communities impacted by floods. 16

Figure S1. Köppen climate classification of all communities. 17

Figure S2. Population density of all communities. 18

Figure S3. Infant mortality rate of all communities. 19

Figure S4. Gross domestic product (GDP) per capita of all communities. 20

References 21

1. Methods

1.1 Hospitalization data

The New South Wales (NSW) Admitted Patient Data Collection (APDC) provided all admissions in all public hospitals, public psychiatric hospitals, multi-purpose services, private hospitals, and private day procedure centers in NSW (containing 32% of the population in Australia), Australia.[1] Hospitalizations financed by the Unified Health System in either public or private hospitals, which account for about 70–80% of hospitalizations in Brazil, were retrieved from the Hospital Information System of the Unified Health System (SIH-SUS).[2] The hospitalization data of Canada were obtained from the Hospital Morbidity Database (HMDB), which provided a complete coverage of all hospital inpatient events in Canada for each second-level administrative divisions (regions or districts within the provinces and territories).[3] The Ministry of Health (MOH) provided all hospitalization records from public hospitals, about 70% of the hospitals in Chile.[4] The hospitalization data were provided by the National Minimum Dataset and covered all admissions records in both public and private hospitals across New Zealand.[5] The hospitalization data of Taiwan sourced by the National Health Insurance Research Database (NHIRD), which covered the hospitalizations from the entire population of Taiwan.[6] However, only the authority provided records with a residential address in the six special municipalities (Taipei, New Taipei, Taoyuan, Taichung, Tainan, and Kaohsiung), which were also the most populous cities in Taiwan to avoid small numbers of daily counts of hospitalization to protect privacy. All hospitalization data across Thailand were obtained from the Ministry of Public Health of Thailand. The hospitalization data of Vietnam were collected from provincial-/city-level hospitals and covered all hospitalizations in 26 provinces. Patients admitted to a hospital outside of the residential province were excluded.[7]

1.2 Demographic and socioeconomic status variables

Population count data in 2010 and 2015 were obtained from the Gridded Population of the World (GPW, version 4) dataset from the Socioeconomic Data And Applications Center (SEDAC), which were estimates of population counts in a spatial resolution of 1km and were consistent with national censuses and population registers.[8] Based on the gridded population data and community boundaries, total population in 2010 were estimated for each community. For each community, population density was defined as number of persons per square kilometer.

Infant mortality rate (IMR) is regarded as an indicator of socioeconomic status (SES).[9] Global estimates of subnational IMR were available in the SEDAC. Only estimates of 2000 and 2015 were available, so we used the estimates of 2015 to represent the SES of the communities. IMR were stored in a gridded format with a spatial resolution of 1km. The population-weighted IMR for each community in 2015 was estimated by using the gridded IMR data and the gridded population counts

data in 2015.

Global data of gross domestic product (GDP) per capita in 2010 were collected with a spatial resolution of 5 are-min.[10] With the population count data in 2010, population-weighted GDP per capita in 2010 was estimated for each community.

1.3 Community-specific model

Community-specific flood-hospitalization associations were examined using a time-series Poisson regression as shown:

$$\log(D_{ij}) = cb(Flood_{ij}) + bs(t_j) + bs(doy_j) + dow_j + cb(Temp_{ij}) + cb(Rain_{ij})$$

Where D_{ij} is the hospitalization count in community i on day j; $Flood_{ij}$ is a cross basis function of flood exposure indicator over 0-210 lag days in community i on day j, of which the exposure-response association was modelled with a strata function (strata: not exposed or exposed), and the lag-response association is modeled using a natural cubic spline with four degrees of freedom; t_j is a cubic B spline with three degrees of freedom for the date on day j;[11] doy_j is a cyclic cubic B spline with three equally spaced knots for day of the year; dow_j is a categorical variable for day of week on day j; $Temp_{ij}$ is a cross basis function of daily mean temperature over 0-21 lag days in community i on day j, of which the exposure-response association is modeled with a quadratic B spline with three internal knots placed at the 10th, 75th, and 90th percentiles, and the lag-response association is modeled using a natural cubic B spline with an intercept and three internal knots placed at equally spaced values in the log scale;[12] and $cb(Rain_{ij})$ is a cross basis function of daily precipitation over 0-14 days in community i on day j, of which the exposure-response association is modeled with a quadratic B spline with three internal knots placed at the 10th, 75th, and 90th percentiles, and the lag-response association is modeled using a natural cubic B spline with two knots placed at equally spaced values in the log scale.[13]

1.4 Sensitivity analyses

We undertook sensitivity analyses to examine the robustness of our results. Namely, assuming different maximum lag periods (180 or 240 days) for the impact of floods on hospitalizations; modeling the lag-response association of flood exposure using a natural cubic B spline with different degrees of freedom (DF = 3 or 5); regarding a community was flooded if its population-weighted centroid or the entire community was within flooded areas; employing a longer lag period (210 days) for the impact of temperature. The effect estimates from the sensitivity analysis models were compared with the effect estimates produced by our primary models, using fixed effect meta-regressions.[14]

1.5 Calculation of attributable fraction

First, the number of annual hospitalizations attributable to flood exposure was calculated for each community using the country-level exposure-lag-response estimates.[15] Then, the total number of annual attributable hospitalizations was divided by the total number of hospitalizations to derive the attributable fraction (AF) for each country. The calculation was performed using the following formulas:

$$b\_AF_j = 1 - \exp(-\sum_{l=0}^{L} \beta_{j-l})$$

$$AN_j = n_j * b\_AF_j$$

$$AF = \frac{\sum {AN_i}/{d_i}}{\sum {n_i}/{d_i}}$$

Where: b_AF_j is the attributable fraction of hospitalization due to the flood exposure on day j, with backward approach; j is the day when hospitalizations occur; l is the lag time; L is the maximum lag time; β_{j-l} is the effect estimates associated with flood exposure on day $j - l$; AN_j is the number of hospitalizations attributable to flood exposure on day j; n_j is the registered number of hospitalizations on day j; AN_i is the total attributable hospitalizations to flood exposure in community i in study period; n_i is the total hospitalizations in community i in study period; d_i is the study period (years) of community i.[15]

Consequently, the 95% empirical confidence intervals were calculated for each AF and AN.[15]

2. Results

Table S1. Characteristics of the communities in each country/territory

	Included communities				Number of excluded communities	Official name of the communities
	Number	Area (km^2)[a]	Study period	Flood days per year[a]		
Australia	84	310 (56 to 6331)	2001-2019	2.6 (2.1 to 4.9)	5	Statistical area level-3
Brazil	422	6014 (3098 to 11629)	2000-2019	3.6 (2.2 to 5.1)	88	Immediate region
Canada	115	2747 (1038 to 10382)	2005-2019	0.7 (0.5 to 1.3)	144	Second-level administrative division
Chile	12	31727 (16004 to 40180)	2001-2019	1.6 (1.4 to 2.3)	3	Region
New Zealand	53	2622 (1269 to 5159)	2000-2019	0.7 (0.5 to 1.1)	13	Territorial authority
Taiwan	6	2146 (1447 to 2244)	2000-2018	2.4 (2.1 to 2.5)	0	Municipality
Thailand	38	4422 (2234 to 5898)	2015-2019	6.4 (5.0 to 8.6)	39	Province
Vietnam	17	3398 (2360 to 5952)	2002-2015	13.5 (1.9 to 17.8)	9	Province

Notes: [a]Median (25th percentile to 75th percentile) of the variable across the included communities during the study period.

Table S2. Number of hospitalizations in all communities during the study period and by country/territory.

	All-cause	CVD	Respiratory	Infectious	Digestive	Mental	Diabetes	Injury	Cancer	Nervous	Renal
Overall	300470192	25875066	27990431	13514768	29032216	7979065	2404337	22709533	13881845	4536011	4000196
Country/territory											
Australia	45953250	2732066	2104960	643290	4678160	2076954	223496	2799396	1877742	1041896	320385
Brazil	119477813	12325102	13365033	8619539	11094001	3354036	1339414	10797481	5433459	1769404	2063697
Canada	35324750	2741851	1993572	421896	3725535	978294	170200	2084316	1516556	428061	276633
Chile	28368408	2099810	2901718	771301	3513785	523536	335186	2675586	1269422	464699	515467
New Zealand	18353266	1356962	1283961	439722	1492142	421478	96700	1736044	835351	389226	105415
Taiwan	36540348	2860406	3842980	626488	2993818	322021	30674	2616710	2392179	237849	338077
Thailand	12480507	1178048	1907207	1591029	1178940	279968	208667	NA	557136	204876	380522
Vietnam	3971850	580821	591000	401503	355835	22778	NA	NA	NA	NA	NA

Abbreviations: NA = not available; CVD = cardiovascular diseases.

Table S3. Cumulative relative risks with 95% confidence interval of hospitalization in 210 days post flood exposure in all communities and by country/territory.

	All-cause	CVD	Respiratory	Infectious	Digestive	Mental	Diabetes	Injury	Cancer	Nervous	Renal
Overall	1.26 (1.15, 1.38)	1.35 (1.21, 1.50)	1.30 (1.13, 1.49)	1.26 (1.10, 1.44)	1.30 (1.17, 1.45)	1.11 (0.98, 1.25)	1.61 (1.39, 1.86)	1.35 (1.21, 1.50)	1.34 (1.21, 1.48)	1.34 (1.20, 1.50)	1.40 (1.22, 1.60)
Australia	0.94 (0.91, 0.98)	0.97 (0.91, 1.04)	1.35 (1.21, 1.49)	1.36 (1.22, 1.51)	0.84 (0.79, 0.89)	0.69 (0.57, 0.83)	5.06 (3.11, 8.25)	1.30 (1.15, 1.46)	1.11 (1.02, 1.21)	1.22 (1.04, 1.44)	0.76 (0.52, 1.12)
Brazil	1.29 (1.14, 1.46)	1.48 (1.30, 1.69)	1.34 (1.11, 1.61)	1.20 (1.00, 1.43)	1.39 (1.21, 1.61)	1.10 (0.95, 1.28)	1.33 (1.12, 1.58)	1.27 (1.11, 1.44)	1.35 (1.19, 1.54)	1.38 (1.17, 1.64)	1.54 (1.30, 1.81)
Canada	2.56 (1.72, 3.80)	2.29 (1.39, 3.78)	2.37 (1.47, 3.82)	2.26 (1.27, 4.02)	2.28 (1.36, 3.81)	1.74 (1.02, 2.94)	1.79 (1.08, 2.97)	2.49 (1.52, 4.08)	2.45 (1.41, 4.24)	1.38 (0.82, 2.31)	2.32 (1.26, 4.30)
Chile	0.99 (0.67, 1.47)	1.04 (0.71, 1.52)	0.50 (0.24, 1.02)	3.93 (1.22, 12.66)	0.87 (0.48, 1.58)	0.99 (0.53, 1.83)	1.17 (0.84, 1.62)	1.87 (1.15, 3.06)	0.98 (0.65, 1.47)	1.53 (0.87, 2.69)	1.13 (0.66, 1.95)
New Zealand	0.96 (0.74, 1.25)	0.91 (0.51, 1.62)	1.50 (0.91, 2.50)	0.80 (0.40, 1.62)	1.10 (0.80, 1.52)	1.55 (0.68, 3.49)	0.62 (0.21, 1.77)	0.76 (0.44, 1.29)	1.40 (0.89, 2.22)	1.70 (0.96, 3.01)	1.92 (0.71, 5.20)
Taiwan	1.43 (1.13, 1.83)	1.45 (0.91, 2.30)	3.23 (2.33, 4.47)	1.01 (0.29, 3.56)	1.35 (0.75, 2.42)	3.67 (1.54, 8.77)	4.24 (0.95, 18.86)	1.94 (1.11, 3.39)	2.15 (1.10, 4.19)	4.52 (1.70, 12.07)	6.77 (3.98, 11.52)
Thailand	0.77 (0.55, 1.09)	0.81 (0.51, 1.27)	0.33 (0.22, 0.51)	1.47 (0.90, 2.39)	0.92 (0.64, 1.35)	1.05 (0.72, 1.53)	1.24 (0.89, 1.74)	NA	0.95 (0.66, 1.37)	1.05 (0.81, 1.37)	1.10 (0.80, 1.52)
Vietnam	0.91 (0.80, 1.03)	1.02 (0.91, 1.16)	0.85 (0.75, 0.97)	0.63 (0.38, 1.06)	1.08 (0.94, 1.23)	1.10 (0.83, 1.45)	NA	NA	NA	NA	NA

Abbreviations: NA = not available; CVD = cardiovascular diseases.

Table S4. Results of sensitivity analyses changing maximum lag period and degrees of freedom of lag-response association.

Model	All-cause	CVD	Respiratory	Infectious	Digestive	Mental	Diabetes	Injury	Cancer	Nervous	Renal
**Primary (ref)**	1.26 (1.15, 1.38)	1.35 (1.21, 1.50)	1.30 (1.13, 1.49)	1.26 (1.10, 1.44)	1.30 (1.17, 1.45)	1.11 (0.98, 1.25)	1.61 (1.39, 1.86)	1.35 (1.21, 1.50)	1.34 (1.21, 1.48)	1.34 (1.20, 1.50)	1.40 (1.22, 1.60)
**Degrees of freedom**											
3	1.26 (1.14, 1.38)	1.34 (1.20, 1.49)	1.29 (1.12, 1.47)	1.24 (1.09, 1.43)	1.29 (1.16, 1.44)	1.08 (0.96, 1.22)	1.56 (1.34, 1.81)	1.34 (1.20, 1.49)	1.32 (1.19, 1.46)	1.31 (1.17, 1.47)	1.36 (1.19, 1.55)
5	1.26 (1.15, 1.39)	1.36 (1.22, 1.51)	1.31 (1.14, 1.50)	1.28 (1.12, 1.47)	1.31 (1.18, 1.47)	1.14 (1.01, 1.29)	1.66 (1.43, 1.93)	1.36 (1.22, 1.52)	1.36 (1.23, 1.50)	1.38 (1.24, 1.54)	1.44 (1.26, 1.65)
6	1.27 (1.15, 1.39)	1.37 (1.23, 1.52)	1.32 (1.15, 1.51)	1.30 (1.13, 1.49)	1.33 (1.19, 1.48)	1.17 (1.03, 1.32)	1.70 (1.47, 1.98)	1.38 (1.23, 1.53)	1.38 (1.25, 1.52)	1.41 (1.27, 1.58)	1.48 (1.29, 1.69)
**Maximum lag period**											
160	1.27 (1.17, 1.37)	1.34 (1.22, 1.46)	1.38 (1.24, 1.54)	1.31 (1.16, 1.48)	1.32 (1.21, 1.45)	1.05 (0.96, 1.16)	1.62 (1.43, 1.85)	1.39 (1.26, 1.52)	1.32 (1.21, 1.44)	1.42 (1.29, 1.56)	1.40 (1.25, 1.58)
180	1.26 (1.16, 1.38)	1.34 (1.22, 1.48)	1.35 (1.19, 1.52)	1.27 (1.12, 1.44)	1.30 (1.18, 1.44)	1.06 (0.96, 1.18)	1.65 (1.44, 1.89)	1.38 (1.25, 1.53)	1.31 (1.19, 1.43)	1.40 (1.26, 1.55)	1.39 (1.23, 1.58)
240	1.27 (1.15, 1.41)	1.38 (1.23, 1.54)	1.31 (1.13, 1.53)	1.26 (1.08, 1.46)	1.36 (1.22, 1.53)	1.15 (1.00, 1.32)	1.62 (1.38, 1.90)	1.35 (1.20, 1.51)	1.42 (1.28, 1.58)	1.37 (1.21, 1.55)	1.36 (1.18, 1.58)
260	1.23 (1.11, 1.36)	1.31 (1.17, 1.47)	1.32 (1.13, 1.55)	1.16 (0.99, 1.37)	1.24 (1.11, 1.40)	1.11 (0.96, 1.29)	1.53 (1.30, 1.80)	1.30 (1.16, 1.47)	1.33 (1.20, 1.47)	1.32 (1.16, 1.49)	1.29 (1.10, 1.51)
**Exposure assessment**											
Population-weighted centroid	1.20 (1.09, 1.32)	1.31 (1.18, 1.46)	1.20 (1.05, 1.38)	1.22 (1.05, 1.40)	1.27 (1.15, 1.42)	1.09 (0.97, 1.23)	1.52 (1.32, 1.75)	1.30 (1.16, 1.45)	1.29 (1.17, 1.43)	1.35 (1.21, 1.50)	1.40 (1.22, 1.60)
Entire community	1.37 (1.19, 1.58)	1.56 (1.33, 1.84)	1.34 (1.11, 1.62)	1.32 (1.08, 1.61)	1.46 (1.25, 1.70)	1.24 (0.99, 1.55)	1.96 (1.57, 2.44)	1.65 (1.41, 1.94)*	1.48 (1.26, 1.73)	1.56 (1.32, 1.85)	1.51 (1.22, 1.88)
**Lag period of temperature**											
210	1.23 (1.10, 1.39)	1.26 (1.12, 1.42)	1.13 (0.97, 1.31)	1.29 (1.09, 1.52)	1.25 (1.11, 1.42)	1.07 (0.93, 1.24)	1.62 (1.36, 1.93)	1.28 (1.13, 1.45)	1.21 (1.08, 1.35)	1.28 (1.13, 1.45)	1.32 (1.13, 1.55)

Note: In the primary model, the lag-response association was model with a maximum lag period of 210 days and 4 degrees of freedom, and a community was flooded if its centroid was within the flooded areas. *, **, and *** represent that p-value for difference were <0.05, <0.01, and <0.001 respectively for the estimate, compared with the estimate of primary analysis. *P*-values for difference between the estimates of primary analyses and sensitivity analyses were calculated through fixed effect meta-regression models.

Table S5. I^2 statistics of pooled effect estimates among communities.

	All-cause	CVD	Respiratory	Infectious	Digestive	Mental	Diabetes	Injury	Cancer	Nervous	Renal
Overall	76%	65%	84%	86%	63%	44%	53%	65%	40%	36%	55%
Country/territory											
Australia	0%	16%	49%	41%	0%	56%	65%	52%	0%	35%	63%
Brazil	81%	67%	86%	87%	67%	33%	48%	69%	46%	37%	55%
Canada	68%	60%	60%	45%	60%	45%	27%	59%	41%	21%	33%
Chile	56%	48%	89%	79%	67%	36%	0%	66%	10%	44%	38%
New Zealand	22%	50%	61%	48%	9%	45%	0%	44%	0%	2%	21%
Taiwan	11%	29%	85%	92%	66%	35%	45%	65%	39%	54%	11%
Thailand	83%	84%	92%	95%	80%	62%	49%	NA	57%	47%	59%
Vietnam	62%	58%	75%	91%	35%	0%	NA	NA	NA	NA	NA
Sex											
Male (ref)	77%	58%	78%	81%	56%	42%	43%	52%	38%	33%	50%
Female	68%	57%	79%	78%	56%	45%	37%	59%	37%	33%	37%
Age, years											
<20	68%	53%	66%	73%	56%	39%	30%	55%	44%	25%	42%
20-60 (ref)	82%	79%	84%	84%	48%	59%	98%	45%	85%	26%	72%
>60	60%	59%	69%	60%	46%	34%	50%	46%	35%	32%	37%
Climate											
Tropical (ref)	85%	74%	89%	91%	72%	43%	50%	71%	50%	40%	60%
Arid	86%	71%	86%	86%	74%	40%	59%	71%	47%	52%	47%
Temperate	33%	44%	74%	73%	43%	43%	57%	57%	22%	32%	51%
Continental	70%	62%	60%	45%	62%	46%	21%	61%	43%	24%	35%
Polar	NA	NA	NA	NA	NA	NA	NA	NA	NA	NA	NA
Population density (per km^2)											
T1: <21 (ref)	74%	53%	75%	81%	55%	33%	46%	56%	24%	30%	49%
T2: 21-65	77%	66%	86%	86%	64%	40%	50%	68%	47%	34%	54%
T3: >65	75%	70%	86%	88%	67%	53%	57%	69%	43%	42%	59%
Infant mortality rate (per 1000 live births)											
T1: <13 (ref)	77%	63%	82%	83%	60%	49%	56%	62%	37%	37%	56%
T2: >13	74%	65%	85%	88%	65%	35%	48%	67%	42%	33%	53%
GDP per capita (1000 USD)											
T1: <20 (ref)	84%	71%	89%	90%	70%	40%	49%	69%	50%	38%	56%

T2: >20	49%	56%	74%	72%	54%	48%	55%	60%	29%	34%	53%
Flood severity											
Lower (ref)	68%	60%	82%	84%	57%	38%	51%	62%	35%	35%	54%
Higher	68%	60%	82%	84%	57%	38%	51%	62%	35%	35%	54%

Notes: There was only one community (of Chile) had polar climate type. Abbreviation: CVD = cardiovascular diseases; USD = constant 2011 United States Dollar.

Table S6. Heterogeneities of effect estimates among all communities and communities in each country/territory and by potential modifiers.

	All-cause	CVD	Respiratory	Infectious	Digestive	Mental	Diabetes	Injury	Cancer	Nervous	Renal
Overall	<.001	<.001	<.001	<.001	<.001	<.001	<.001	<.001	<.001	<.001	<.001
Country/territory											
Australia	1	0.008	<.001	<.001	1	<.001	<.001	<.001	0.925	<.001	<.001
Brazil	<.001	<.001	<.001	<.001	<.001	<.001	<.001	<.001	<.001	<.001	<.001
Canada	<.001	<.001	<.001	<.001	<.001	<.001	0.002	<.001	<.001	0.003	<.001
Chile	<.001	<.001	<.001	<.001	<.001	0.01	0.597	<.001	0.284	<.001	0.006
New Zealand	0.004	<.001	<.001	<.001	0.176	<.001	0.518	<.001	0.599	0.43	0.069
Taiwan	0.318	0.102	<.001	<.001	<.001	0.074	0.066	<.001	0.035	0.01	0.321
Thailand	<.001	<.001	<.001	<.001	<.001	<.001	<.001	NA	<.001	<.001	<.001
Vietnam	<.001	<.001	<.001	<.001	0.004	0.924	NA	NA	NA	NA	NA
Sex											
Male (ref)	<.001	<.001	<.001	<.001	<.001	<.001	<.001	<.001	<.001	<.001	<.001
Female	<.001	<.001	<.001	<.001	<.001	<.001	<.001	<.001	<.001	<.001	<.001
Age, years											
<20	<.001	<.001	<.001	<.001	<.001	<.001	<.001	<.001	<.001	<.001	<.001
20-60 (ref)	<.001	<.001	<.001	<.001	<.001	<.001	<.001	<.001	<.001	<.001	<.001
>60	<.001	<.001	<.001	<.001	<.001	<.001	<.001	<.001	<.001	<.001	<.001
Climate											
Tropical (ref)	<.001	<.001	<.001	<.001	<.001	<.001	<.001	<.001	<.001	<.001	<.001
Arid	<.001	<.001	<.001	<.001	<.001	<.001	<.001	<.001	<.001	<.001	<.001
Temperate	<.001	<.001	<.001	<.001	<.001	<.001	<.001	<.001	<.001	<.001	<.001
Continental	<.001	<.001	<.001	<.001	<.001	<.001	0.021	<.001	<.001	<.001	<.001
Polar	NA	NA	NA	NA	NA	NA	NA	NA	NA	NA	NA
Population density (per km2)											
T1: <21 (ref)	<.001	<.001	<.001	<.001	<.001	<.001	<.001	<.001	<.001	<.001	<.001
T2: 21-65	<.001	<.001	<.001	<.001	<.001	<.001	<.001	<.001	<.001	<.001	<.001
T3: >65	<.001	<.001	<.001	<.001	<.001	<.001	<.001	<.001	<.001	<.001	<.001
Infant mortality rate (per 1000 live births)											
T1: <13 (ref)	<.001	<.001	<.001	<.001	<.001	<.001	<.001	<.001	<.001	<.001	<.001
T2: >13	<.001	<.001	<.001	<.001	<.001	<.001	<.001	<.001	<.001	<.001	<.001
GDP per capita (1000 USD)											

T1: <20 (ref)	<.001	<.001	<.001	<.001	<.001	<.001	<.001	<.001	<.001	<.001	<.001
T2: >20	<.001	<.001	<.001	<.001	<.001	<.001	<.001	<.001	<.001	<.001	<.001
Flood severity											
Lower (ref)	<.001	<.001	<.001	<.001	<.001	<.001	<.001	<.001	<.001	<.001	<.001
Higher	<.001	<.001	<.001	<.001	<.001	<.001	<.001	<.001	<.001	<.001	<.001

Note: *p*-values were estimated through Cochran's Q tests. There was only one community (of Chile) had polar climate type. Abbreviation: CVD = cardiovascular diseases; USD = constant 2011 United States Dollar.

Table S7. Cumulative relative risks of hospitalization in 210 days post flood exposure by potential modifiers.

	All-cause	CVD	Respiratory	Infectious	Digestive	Mental	Diabetes	Injury	Cancer	Nervous	Renal
Sex											
Male (ref)	1.30 (1.18, 1.43)	1.31 (1.18, 1.47)	1.29 (1.12, 1.49)	1.26 (1.08, 1.46)	1.32 (1.18, 1.48)	1.26 (1.07, 1.47)	1.52 (1.29, 1.80)	1.36 (1.21, 1.53)	1.43 (1.28, 1.60)	1.36 (1.20, 1.54)	1.42 (1.20, 1.67)
Female	1.24 (1.13, 1.36)	1.41 (1.26, 1.57)	1.35 (1.17, 1.55)	1.34 (1.17, 1.54)	1.33 (1.19, 1.48)	1.06 (0.91, 1.22)	1.89 (1.61, 2.22)	1.39 (1.24, 1.55)	1.30 (1.16, 1.46)	1.46 (1.27, 1.67)	1.51 (1.31, 1.74)
Age, years											
<20	1.23 (1.10, 1.37)	1.31 (0.87, 1.98)	1.15 (0.96, 1.37)	1.10 (0.93, 1.31)*	1.40 (1.22, 1.60)	1.60 (1.19, 2.16)**	5.80 (0.81, 41.43)	1.33 (1.17, 1.51)	2.52 (0.67, 9.44)	1.63 (1.37, 1.93)*	4.10 (1.30, 12.90)
20-60 (ref)	1.26 (1.15, 1.39)	1.34 (1.19, 1.52)	1.18 (1.02, 1.37)	1.48 (1.26, 1.74)	1.28 (1.14, 1.43)	1.00 (0.86, 1.16)	1.43 (1.19, 1.71)	1.30 (1.16, 1.44)	1.43 (1.26, 1.63)	1.28 (1.12, 1.46)	1.54 (1.32, 1.79)
>60	1.31 (1.18, 1.44)	1.37 (1.23, 1.53)	1.49 (1.31, 1.70)*	1.55 (1.34, 1.78)	1.37 (1.23, 1.53)	1.15 (0.92, 1.44)	1.89 (1.52, 2.35)	1.58 (1.40, 1.80)*	1.36 (1.22, 1.51)	1.74 (1.48, 2.05)**	1.37 (1.17, 1.59)
Climate											
Tropical (ref)	1.33 (1.13, 1.57)	1.61 (1.33, 1.95)	1.36 (1.05, 1.75)	1.25 (0.98, 1.57)	1.45 (1.20, 1.75)	1.35 (1.04, 1.74)	1.58 (1.25, 2.00)	1.47 (1.19, 1.82)	1.43 (1.18, 1.73)	1.41 (1.12, 1.76)	1.61 (1.28, 2.03)
Arid	1.96 (1.22, 3.17)	1.83 (1.08, 3.12)	4.92 (2.52, 9.58)***	3.72 (1.95, 7.09)**	1.63 (0.89, 2.97)	0.70 (0.40, 1.22)*	2.00 (0.86, 4.66)	1.76 (1.04, 2.98)	3.29 (1.80, 6.02)*	1.91 (0.89, 4.13)	2.44 (1.25, 4.77)
Temperate	0.97 (0.93, 1.02)***	1.02 (0.94, 1.10)***	0.97 (0.86, 1.09)*	1.09 (0.93, 1.28)	1.01 (0.92, 1.10)**	0.93 (0.82, 1.05)*	1.58 (1.29, 1.94)	1.10 (1.00, 1.21)*	1.10 (1.02, 1.18)*	1.26 (1.13, 1.40)	1.12 (0.94, 1.33)*
Continental	2.65 (1.75, 4.01)**	2.38 (1.41, 4.02)	2.24 (1.37, 3.67)	2.23 (1.23, 4.05)	2.48 (1.45, 4.23)	1.88 (1.08, 3.29)	1.26 (0.94, 1.68)	2.60 (1.55, 4.38)*	2.51 (1.41, 4.45)	1.40 (0.82, 2.41)	2.48 (1.33, 4.65)
Polar	1.10 (0.71, 1.71)	1.47 (0.93, 2.32)	0.95 (0.62, 1.47)	0.54 (0.29, 0.99)*	1.68 (1.04, 2.73)	1.36 (0.65, 2.86)	0.87 (0.38, 1.99)	1.15 (0.78, 1.70)	0.68 (0.25, 1.88)	1.29 (0.58, 2.86)	0.71 (0.35, 1.47)*
Population density (per km^2)											
<21 (ref)	1.04 (0.89, 1.22)	1.20 (1.01, 1.42)	1.48 (1.17, 1.87)	1.03 (0.79, 1.36)	0.89 (0.73, 1.09)	1.03 (0.84, 1.27)	1.43 (1.07, 1.92)	1.07 (0.90, 1.28)	1.12 (0.96, 1.31)	1.10 (0.88, 1.37)	1.40 (1.08, 1.82)
21-65	1.37 (1.17, 1.60)*	1.45 (1.21, 1.74)	1.20 (0.95, 1.53)	1.26 (0.99, 1.60)	1.47 (1.24, 1.73)***	1.11 (0.91, 1.36)	1.33 (1.05, 1.69)	1.25 (1.05, 1.47)	1.36 (1.15, 1.60)	1.43 (1.19, 1.71)	1.44 (1.16, 1.78)
>65	1.38 (1.16, 1.64)*	1.38 (1.15, 1.67)	1.28 (1.02, 1.61)	1.46 (1.20, 1.79)*	1.55 (1.28, 1.87)***	1.11 (0.90, 1.36)	2.15 (1.68, 2.76)*	1.91 (1.54, 2.38)***	1.46 (1.22, 1.76)*	1.43 (1.20, 1.70)	1.33 (1.06, 1.67)
Infant mortality rate (per 1000 live births)											
<13 (ref)	1.32 (1.13,	1.28 (1.07,	1.58 (1.27,	1.44 (1.18,	1.25 (1.04,	1.15 (0.96,	2.42 (1.86,	1.48 (1.23,	1.43 (1.21,	1.33 (1.13,	1.36 (1.07,

	1.55)	1.53)	1.96)	1.76)	1.49)	1.39)	3.17)	1.77)	1.69)	1.57)	1.72)
>13	1.17 (1.05, 1.29)	1.38 (1.22, 1.56)	1.10 (0.92, 1.30)*	1.10 (0.91, 1.33)	1.31 (1.15, 1.50)	1.03 (0.89, 1.19)	1.23 (1.03, 1.46)***	1.22 (1.07, 1.39)	1.25 (1.10, 1.40)	1.36 (1.17, 1.57)	1.43 (1.21, 1.68)
GDP per capita (1000 USD)											
<20 (ref)	1.24 (1.09, 1.43)	1.24 (1.08, 1.43)	1.15 (0.94, 1.42)	1.24 (1.02, 1.51)	1.23 (1.05, 1.42)	1.14 (0.97, 1.33)	1.23 (1.03, 1.47)	1.15 (0.99, 1.34)	1.26 (1.09, 1.45)	1.26 (1.06, 1.50)	1.41 (1.18, 1.68)
>20	1.32 (1.15, 1.52)	1.48 (1.25, 1.75)	1.43 (1.22, 1.69)	1.36 (1.13, 1.63)	1.43 (1.22, 1.69)	1.05 (0.87, 1.26)	2.45 (1.91, 3.13)***	1.63 (1.38, 1.92)**	1.44 (1.25, 1.64)	1.38 (1.20, 1.59)	1.41 (1.13, 1.75)
Flood severity											
Lower (ref)	1.31 (1.17, 1.46)	1.71 (1.50, 1.96)	1.46 (1.24, 1.73)	1.17 (0.95, 1.43)	1.47 (1.28, 1.69)	1.31 (1.08, 1.60)	2.89 (2.31, 3.63)	1.65 (1.43, 1.90)	1.59 (1.38, 1.82)	1.78 (1.48, 2.14)	2.18 (1.78, 2.68)
Higher	1.09 (1.03, 1.17)**	1.01 (0.92, 1.12)***	1.06 (0.91, 1.23)**	1.39 (1.13, 1.71)	0.98 (0.89, 1.07)***	0.96 (0.84, 1.09)**	1.26 (1.03, 1.53)***	1.08 (0.97, 1.22)***	1.04 (0.94, 1.16)***	1.19 (1.06, 1.33)***	0.97 (0.80, 1.18)***

Note: There was only one community (of Chile) with polar climate type. *, **, and *** represent that p-value for difference were <0.05, <0.01, and <0.001 respectively for the estimate, compared with the reference group, where the comparison was performed using meta-regression model. Abbreviations: CVD = cardiovascular diseases; ref = reference group; USD = constant 2011 United States Dollar.

Table S8. Attributable fractions (%) of hospitalizations due to flood exposure in communities impacted by floods.

	All-cause	CVD	Respiratory	Infectious	Digestive	Mental	Diabetes	Injury	Cancer	Nervous	Renal
Overall	0.07 (-0.13, 0.28)	0.18 (-0.11, 0.46)	-0.28 (-0.67, 0.09)	0.31 (-0.24, 0.82)	0.14 (-0.12, 0.40)	0.04 (-0.24, 0.32)	0.42 (0.07, 0.76)	0.25 (0.09, 0.40)	0.22 (-0.04, 0.47)	0.28 (0.01, 0.53)	0.37 (0.02, 0.71)
Australia	-0.06 (-0.09, -0.02)	-0.03 (-0.09, 0.04)	0.31 (0.21, 0.42)	0.30 (0.20, 0.40)	-0.17 (-0.23, -0.12)	-0.36 (-0.55, -0.18)	1.93 (1.39, 2.41)	0.25 (0.14, 0.36)	0.10 (0.02, 0.18)	0.20 (0.04, 0.35)	-0.25 (-0.62, 0.10)
Brazil	0.26 (0.14, 0.38)	0.41 (0.27, 0.54)	0.32 (0.12, 0.52)	0.21 (0.02, 0.40)	0.34 (0.20, 0.48)	0.11 (-0.07, 0.29)	0.30 (0.12, 0.47)	0.23 (0.10, 0.36)	0.31 (0.18, 0.44)	0.35 (0.17, 0.53)	0.46 (0.29, 0.63)
Canada	0.27 (0.16, 0.37)	0.26 (0.11, 0.40)	0.25 (0.11, 0.37)	0.25 (0.08, 0.40)	0.26 (0.10, 0.40)	0.19 (0.01, 0.35)	0.22 (0.03, 0.39)	0.28 (0.14, 0.42)	0.26 (0.11, 0.41)	0.13 (-0.08, 0.31)	0.26 (0.07, 0.43)
Chile	0.00 (-0.18, 0.18)	0.02 (-0.16, 0.19)	-0.33 (-0.72, 0.04)	0.57 (0.10, 0.99)	-0.06 (-0.35, 0.21)	0.00 (-0.30, 0.28)	0.07 (-0.08, 0.21)	0.29 (0.07, 0.51)	-0.01 (-0.21, 0.18)	0.21 (-0.08, 0.48)	0.06 (-0.19, 0.30)
New Zealand	-0.01 (-0.07, 0.05)	-0.02 (-0.16, 0.11)	0.10 (-0.03, 0.22)	-0.05 (-0.22, 0.12)	0.02 (-0.05, 0.10)	0.10 (-0.09, 0.29)	-0.13 (-0.44, 0.17)	-0.07 (-0.20, 0.06)	0.08 (-0.02, 0.18)	0.13 (-0.01, 0.27)	0.16 (-0.09, 0.40)
Taiwan	0.24 (0.09, 0.39)	0.25 (-0.07, 0.55)	0.75 (0.54, 0.96)	-0.01 (-0.95, 0.67)	0.21 (-0.20, 0.60)	0.90 (0.26, 1.41)	1.39 (-0.40, 2.75)	0.46 (0.07, 0.82)	0.46 (0.05, 0.85)	1.17 (0.39, 1.81)	1.47 (1.05, 1.88)
Thailand	-0.64 (-1.50, 0.18)	-0.53 (-1.70, 0.58)	-2.78 (-3.98, -1.65)	0.84 (-0.32, 1.90)	-0.19 (-1.15, 0.69)	0.12 (-0.82, 1.02)	0.54 (-0.29, 1.32)	NA	-0.13 (-1.06, 0.76)	0.13 (-0.54, 0.75)	0.23 (-0.54, 0.97)
Vietnam	-0.27 (-0.65, 0.09)	0.09 (-0.35, 0.51)	-0.47 (-0.86, -0.12)	-1.68 (-3.91, 0.23)	0.29 (-0.22, 0.79)	0.57 (-1.06, 2.02)	NA	NA	NA	NA	NA

Notes: Estimates are presented with 95% CI. Abbreviations: NA = not available; CVD = cardiovascular diseases.

Table S9. Annual flood-attributable hospitalizations in communities impacted by floods.

Country	All-cause	CVD	Respiratory	Infectious	Digestive	Mental	Diabetes	Injury	Cancer	Nervous	Renal
Overall	15920 (-28272, 58765)	3422 (-2066, 8656)	-5886 (-14233, 2019)	3734 (-2885, 9755)	2937 (-2481, 8088)	248 (-1340, 1742)	814 (129, 1464)	3651 (1281, 5947)	2091 (-428, 4521)	879 (31, 1673)	1191 (79, 2268)
Australia	-1421 (-2348, -502)	-39 (-134, 55)	357 (237, 476)	105 (70, 139)	-438 (-583, -295)	-406 (-614, -202)	233 (168, 291)	376 (208, 542)	104 (19, 187)	111 (23, 196)	-44 (-107, 17)
Brazil	23797 (12662, 34822)	3801 (2524, 5062)	3308 (1266, 5310)	1424 (142, 2676)	2852 (1649, 4039)	277 (-186, 722)	303 (119, 485)	1898 (852, 2922)	1271 (740, 1793)	457 (225, 684)	726 (450, 994)
Canada	6405 (3850, 8808)	471 (196, 725)	329 (150, 495)	70 (22, 112)	640 (257, 997)	122 (5, 229)	25 (3, 44)	395 (191, 586)	265 (108, 415)	36 (-23, 87)	47 (14, 79)
Chile	-43 (-2768, 2696)	20 (-178, 214)	-498 (-1106, 60)	231 (42, 403)	-119 (-644, 389)	-1 (-84, 78)	13 (-14, 37)	408 (101, 717)	-8 (-140, 118)	51 (-19, 118)	16 (-52, 82)
New Zealand	-87 (-627, 439)	-14 (-108, 76)	62 (-21, 142)	-10 (-48, 26)	17 (-40, 72)	22 (-20, 61)	-6 (-21, 8)	-58 (-170, 53)	33 (-10, 76)	25 (-2, 52)	8 (-5, 21)
Taiwan	4631 (1658, 7590)	377 (-108, 830)	1523 (1088, 1935)	-2 (-312, 220)	331 (-319, 951)	152 (45, 239)	22 (-7, 44)	632 (99, 1128)	574 (58, 1067)	147 (48, 227)	262 (187, 335)
Thailand	-16056 (-37566, 4469)	-1251 (-4035, 1369)	-10637 (-15245, -6313)	2680 (-1031, 6076)	-452 (-2718, 1647)	70 (-462, 571)	224 (-121, 553)	NA	-147 (-1202, 864)	52 (-221, 308)	176 (-409, 742)
Vietnam	-1306 (-3133, 443)	58 (-222, 325)	-330 (-602, -87)	-762 (-1770, 103)	106 (-82, 289)	13 (-24, 45)	NA	NA	NA	NA	NA

Note: Estimates are presented with 95% CI. Abbreviations: NA = not available; CVD = cardiovascular diseases.

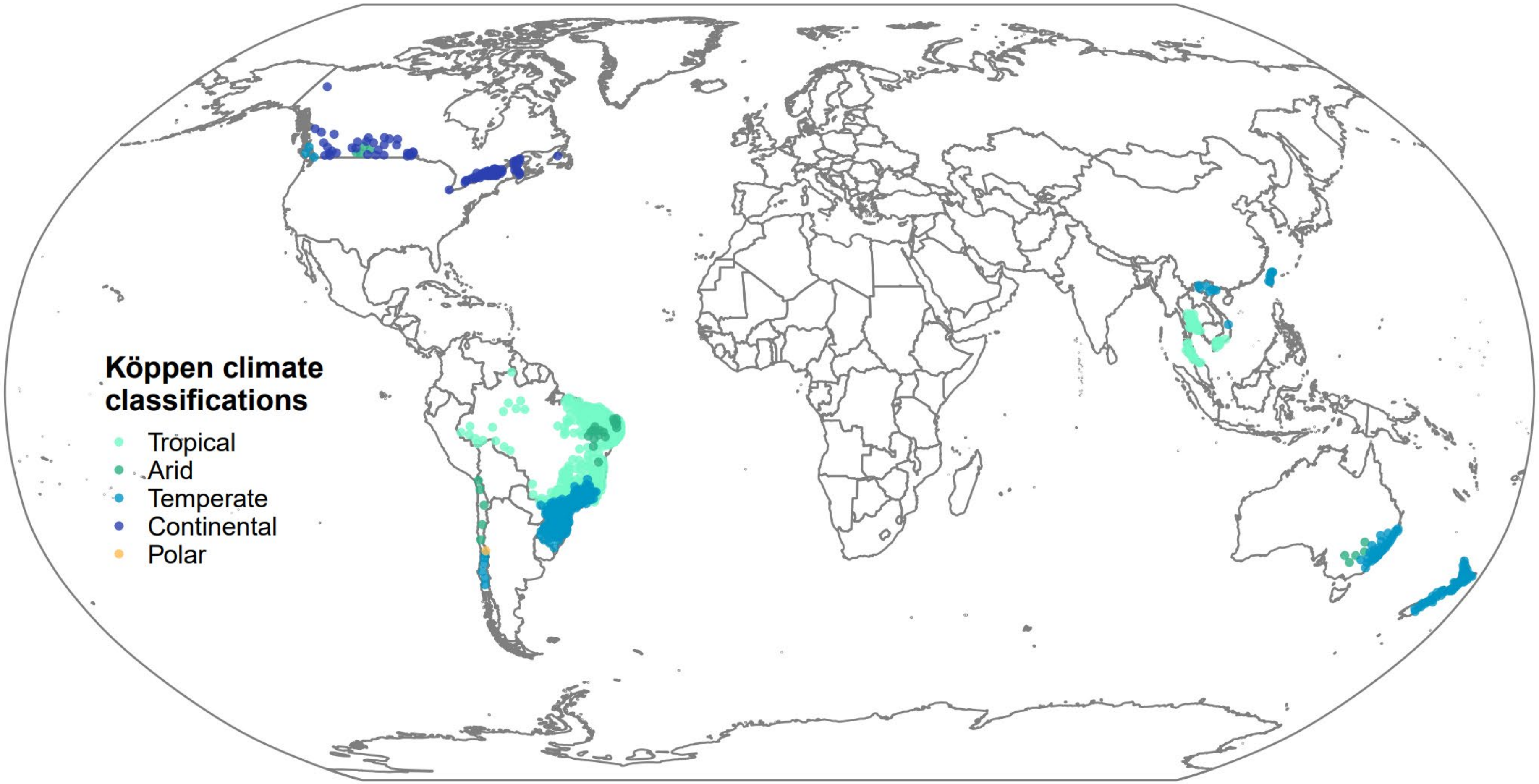

Figure S1. Köppen climate classification of all communities.

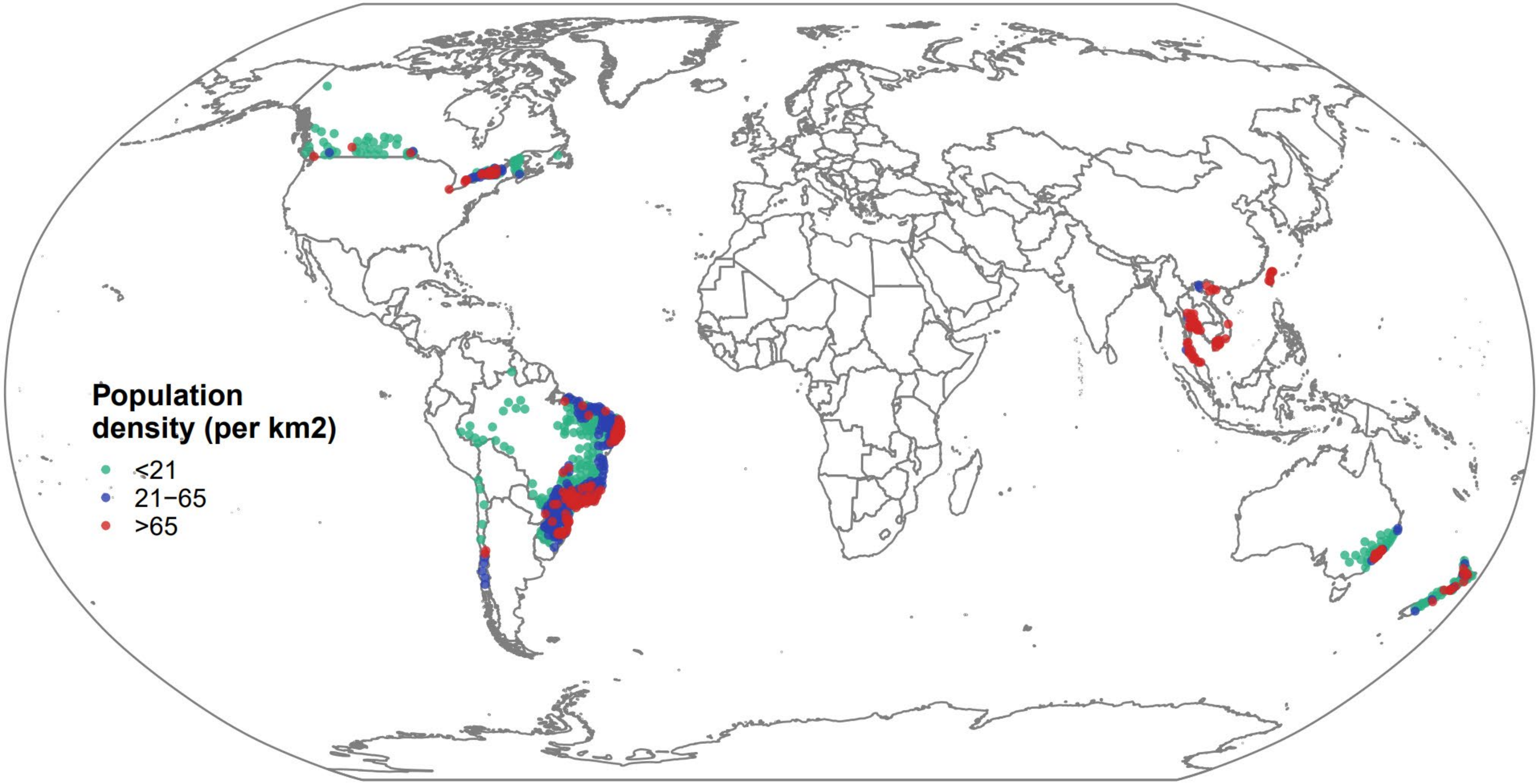

Figure S2. Population density of all communities.

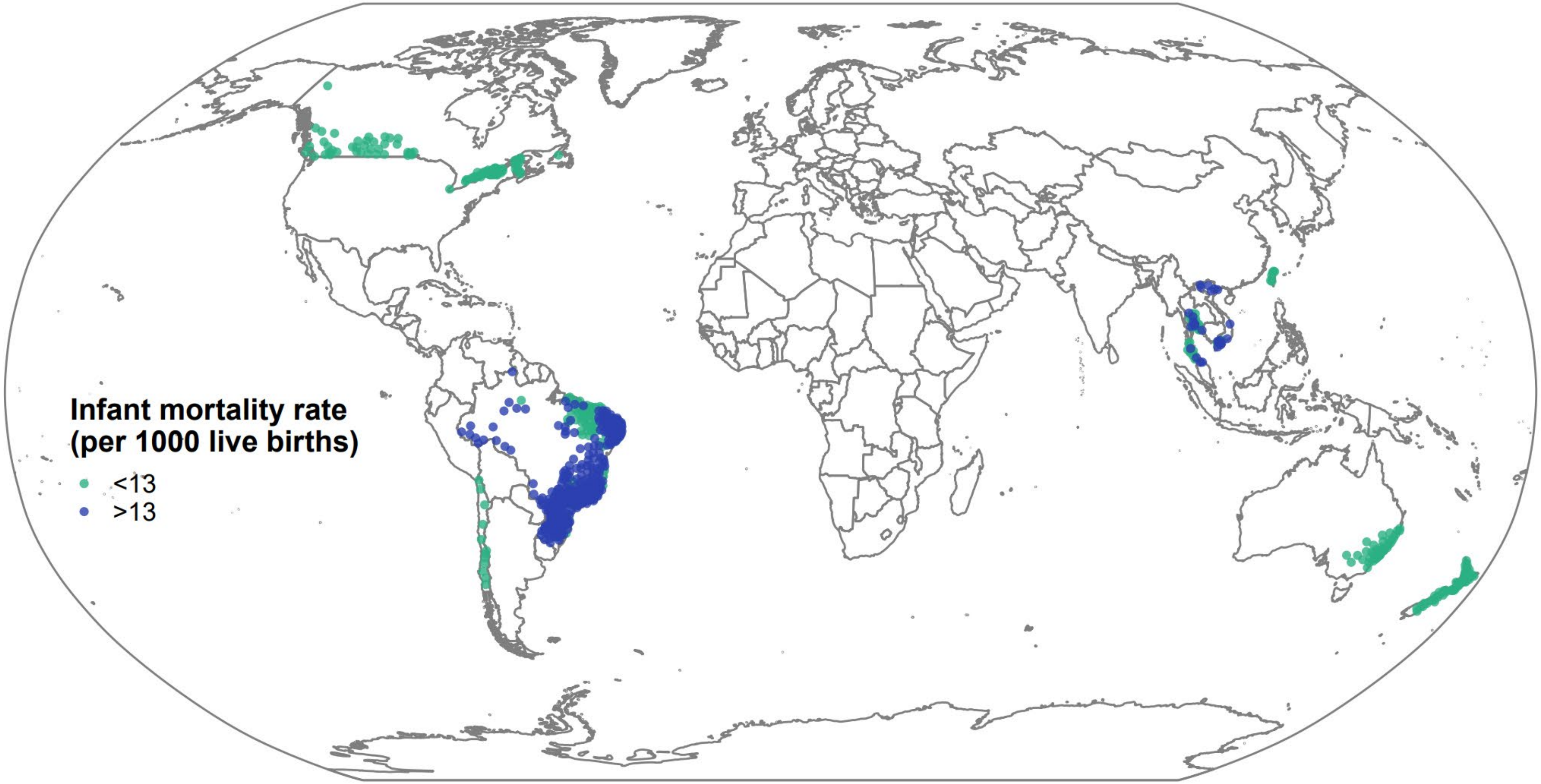

Figure S3. Infant mortality rate of all communities.

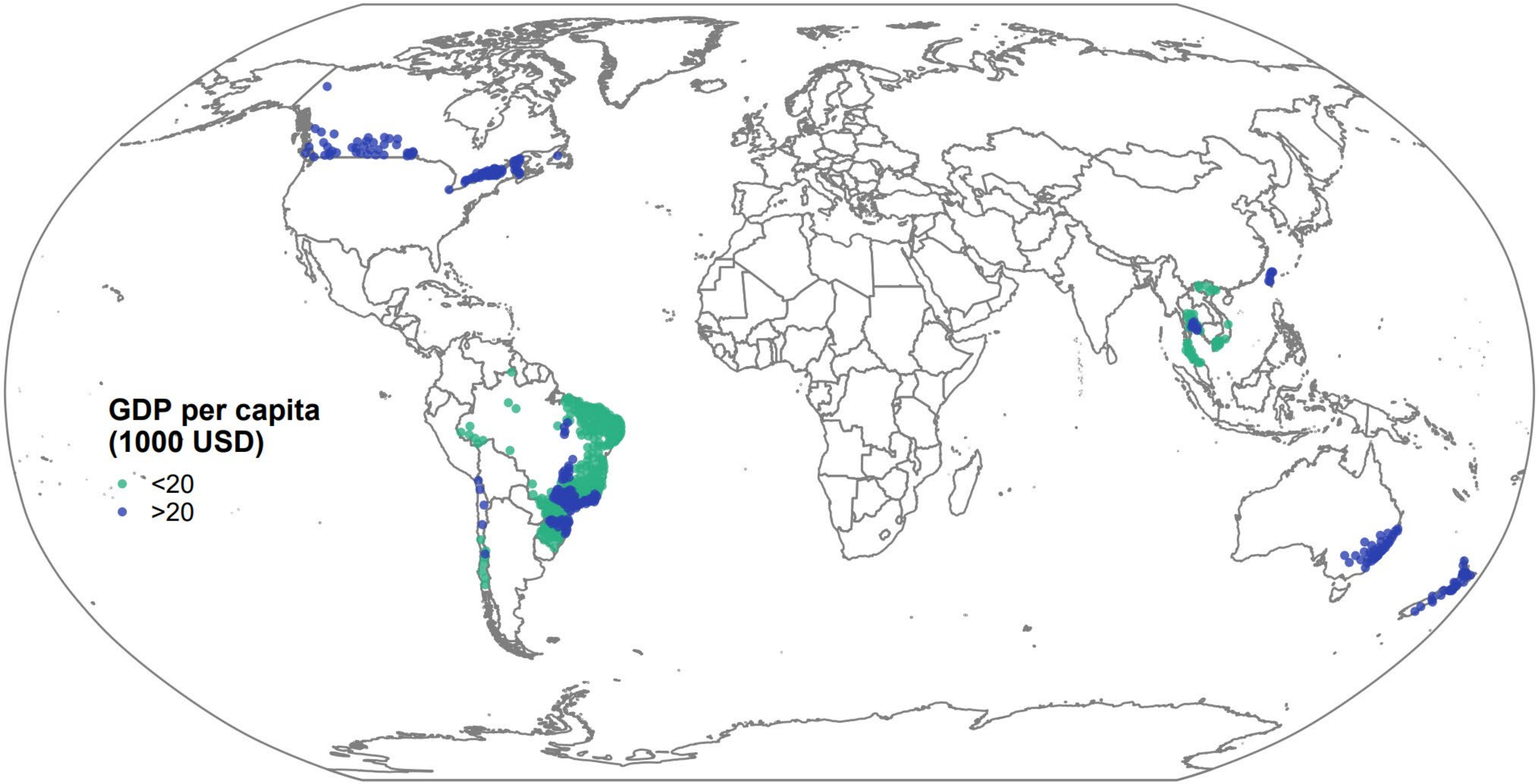

Figure S4. Gross domestic product (GDP) per capita of all communities.

References

1. Lam, M. K. How good is New South Wales admitted patient data collection in recording births? *Health Inf Manag* **40**, 12–19 (2011).
2. Nakamura-Pereira, M., Mendes-Silva, W., Dias, M. A. B., Reichenheim, M. E. & Lobato, G. Sistema de Informações Hospitalares do Sistema Único de Saúde (SIH-SUS): uma avaliação do seu desempenho para a identificação do near miss materno [The Hospital Information System of the Brazilian Unified National Health System: a performance evaluation for auditing maternal near miss]. *Cad Saude Publica* **29**, 1333–1345 (2013).
3. Amuah, J. E. *et al.* Development and validation of a hospital frailty risk measure using Canadian clinical administrative data. *CMAJ* **195**, E437–E448 (2023).
4. Dales, R., Blanco-Vidal, C. & Cakmak, S. The Association Between Air Pollution and Hospitalization of Patients With Idiopathic Pulmonary Fibrosis in Chile: A Daily Time Series Analysis. *Chest* **158**, 630–636 (2020).
5. National Minimum Dataset (Hospital Events): New Zealand Ministry of Health (version 7.9). (2021).
6. Lin, L.-Y., Warren-Gash, C., Smeeth, L. & Chen, P.-C. Data resource profile: the National Health Insurance Research Database (NHIRD). *Epidemiol Health* **40**, e2018062 (2018).
7. Phung, D. *et al.* High temperature and risk of hospitalizations, and effect modifying potential of socio-economic conditions: A multi-province study in the tropical Mekong Delta Region. *Environ Int* **92–93**, 77–86 (2016).
8. Center For International Earth Science Information Network-CIESIN-Columbia University. Gridded Population of the World, Version 4 (GPWv4): Basic Demographic Characteristics, Revision 11. Palisades, NY: NASA Socioeconomic Data and Applications Center (SEDAC) https://doi.org/10.7927/H46M34XX (2018).
9. Gonzalez, R. M. & Gilleskie, D. Infant Mortality Rate as a Measure of a Country's Health: A Robust Method to Improve Reliability and Comparability. *Demography* **54**, 701–720 (2017).
10. Kummu, M., Taka, M. & Guillaume, J. H. A. Gridded global datasets for Gross Domestic Product and Human Development Index over 1990-2015. *Sci Data* **5**, 180004 (2018).

11. Scortichini, M. *et al.* Excess mortality during the COVID-19 outbreak in Italy: a two-stage interrupted time-series analysis. *Int J Epidemiol* **49**, 1909–1917 (2020).
12. Gasparrini, A. *et al.* Mortality risk attributable to high and low ambient temperature: a multicountry observational study. *The Lancet* **386**, 369–375 (2015).
13. He, C. *et al.* Rainfall events and daily mortality across 645 global locations: two stage time series analysis. *BMJ* **387**, e080944 (2024).
14. Borenstein, M., Hedges, L. V., Higgins, J. P. T. & Rothstein, H. R. A basic introduction to fixed-effect and random-effects models for meta-analysis. *Res. Synth. Method* **1**, 97–111 (2010).
15. Gasparrini, A. & Leone, M. Attributable risk from distributed lag models. *BMC Medical Research Methodology* **14**, 55 (2014).
