# Supplementary figures and images for: Hospitalization risks associated with floods in a multi-country study
Source: Nat Water. 2025 Apr 8;3(5):561–70. doi: 10.1038/s44221-025-00425-8 (PMC12098117; doi:10.1038/s44221-025-00425-8)

Flood days  
per year

5 10 15 20 25

**NZL**

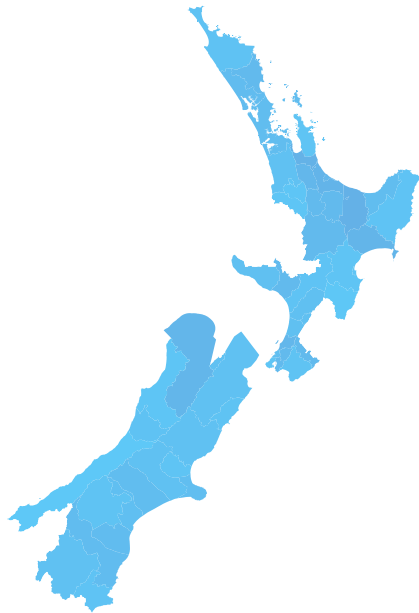

**NSW, AUS**

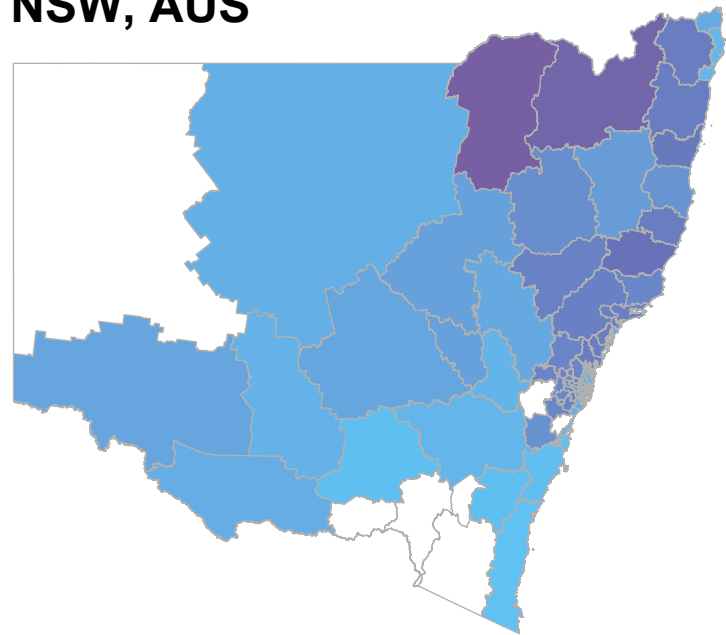

**BRA**

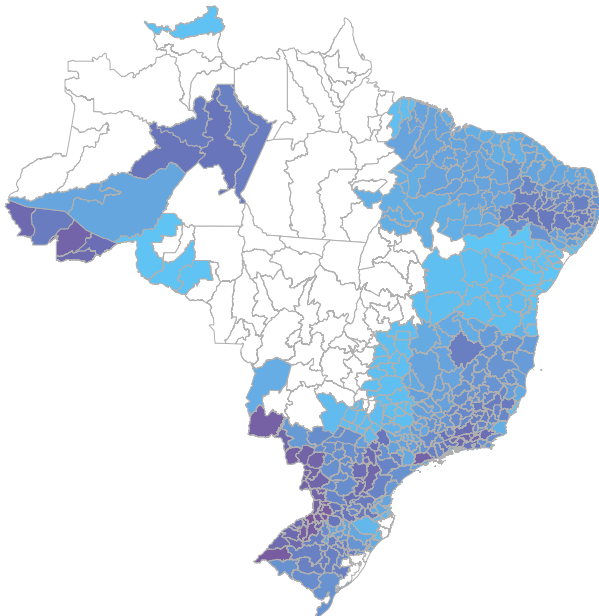

**CAN**

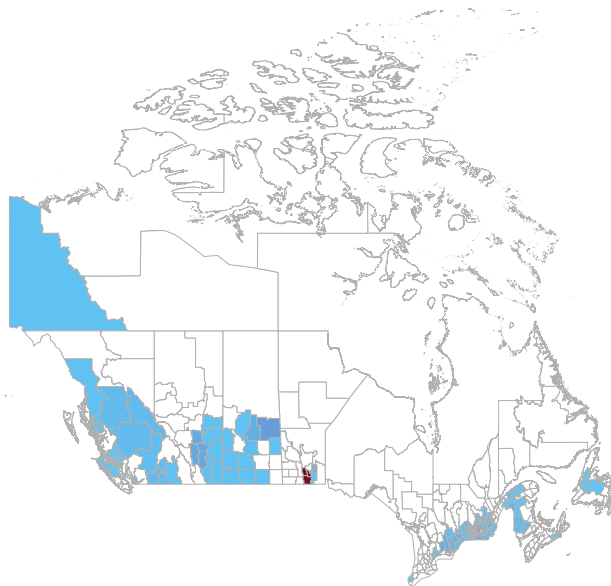

**THL**

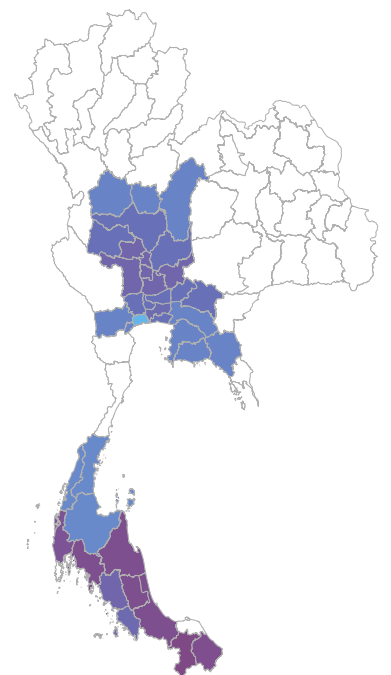

**CHL**

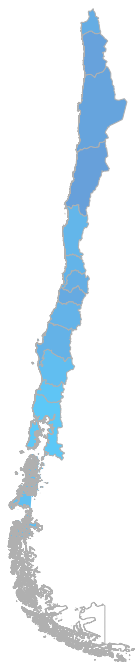

**VNM**

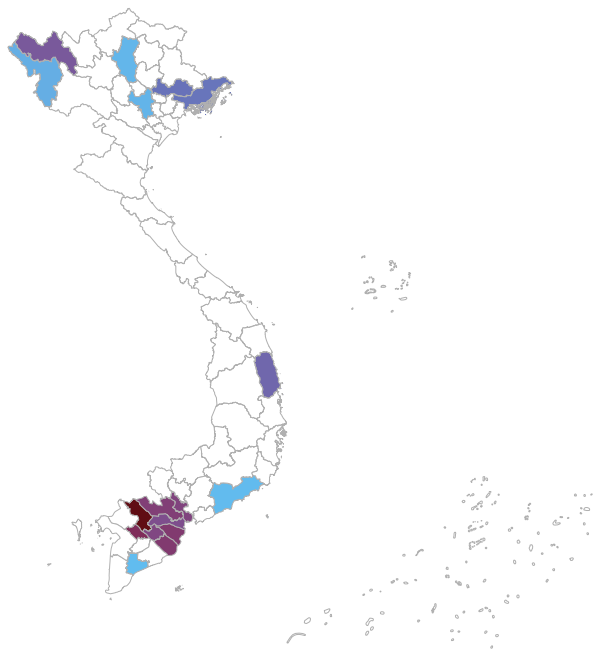

**TWN**

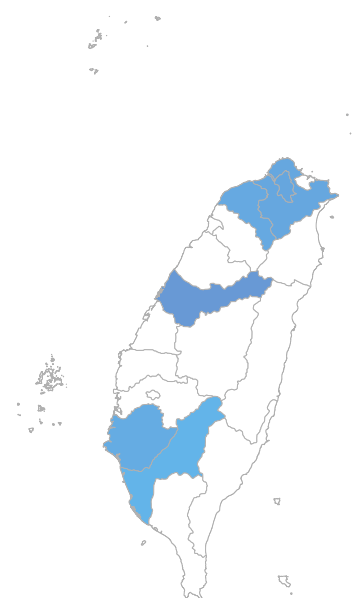

Supplement: Supplementary file 2 — Unprocessed western blots and/or gels. [file 44221_2025_425_MOESM2_ESM.pdf]

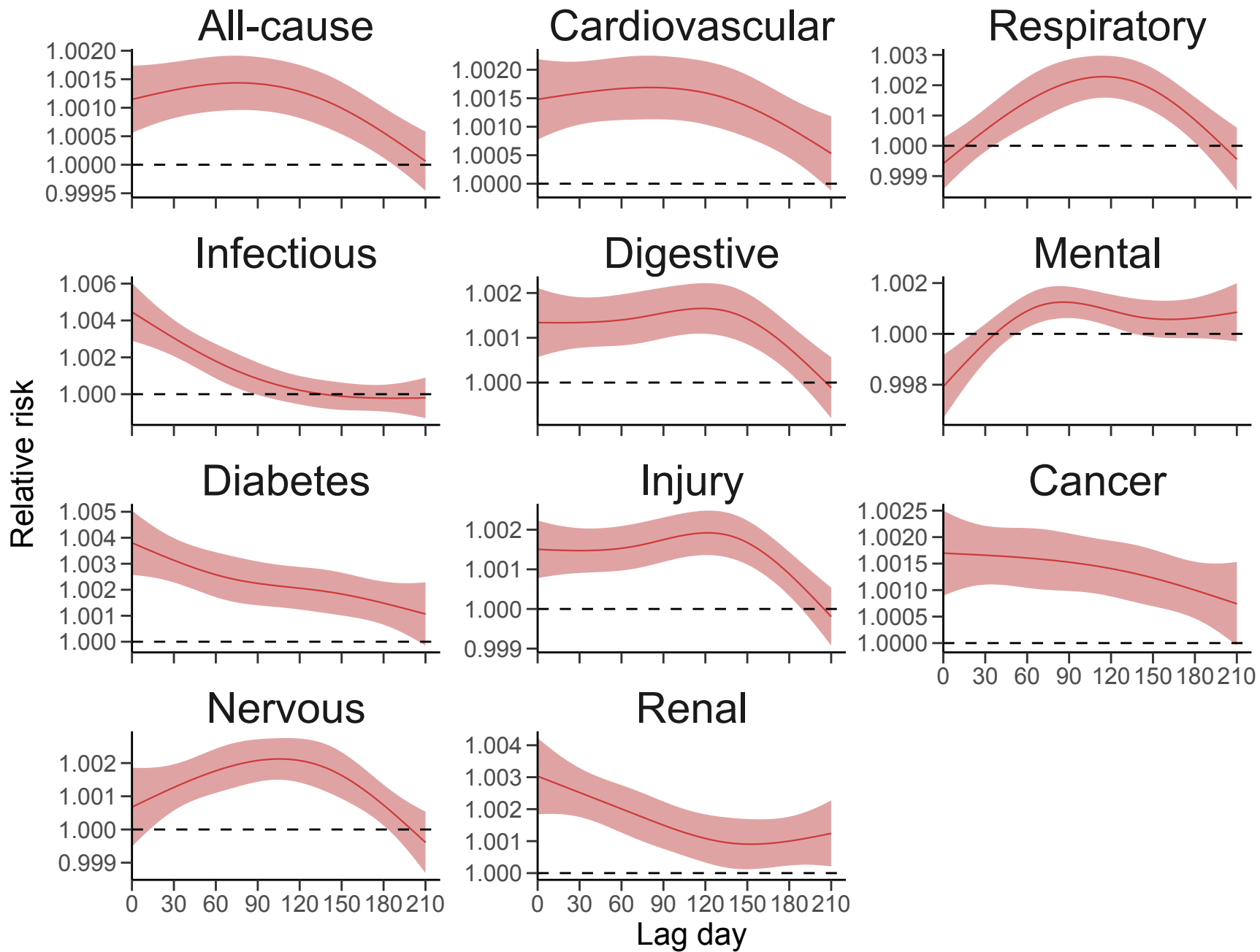

Supplement: Supplementary file 3 — Unprocessed western blots and/or gels. [file 44221_2025_425_MOESM3_ESM.pdf]

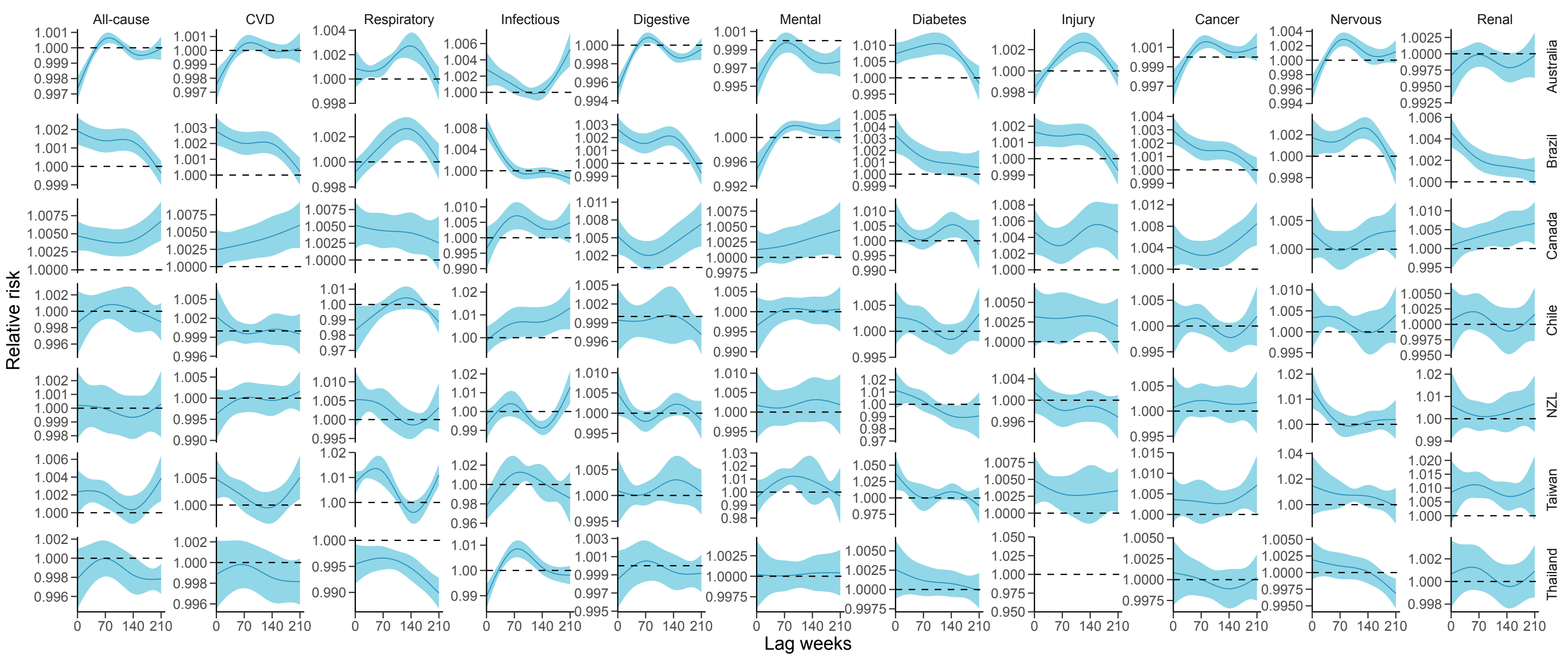

Supplement: Supplementary file 7 — Unprocessed western blots and/or gels. [file 44221_2025_425_MOESM7_ESM.pdf]
